# Dissociable roles of reward prediction error in the contrasting mood dynamics of depression and anxiety

**DOI:** 10.7554/eLife.110631

**Published:** 2026-09-29

**Authors:** Zhihao Wang, Ting Wang, Tian Nan, Jiahua Xu, André Aleman, Yuejia Luo, Bastien Blain, Yunzhe Liu, Pengfei Xu

**Affiliations:** 1 Center for Neurocognition and Social Behavior, Institute of Artificial Intelligence, Shenzhen University of Advanced Technology Shenzhen China; 2 CNRS - Centre d'Economie de la Sorbonne, Panthéon-Sorbonne University Paris France; 3 Institute for brain research and rehabilitation, South China Normal University Guangzhou China; 4 The State Key Lab of Cognitive and Learning, Faculty of Psychology, Beijing Normal University Beijing China; 5 State Key Laboratory of Cognitive Neuroscience and Learning, IDG/McGovern Institute for Brain Research, Beijing Normal University Beijing China; 6 Chinese Institute for Brain Research Beijing China; 7 Faculty of Psychology and Neuroscience, Maastricht University Maastricht Netherlands; 8 Institute for Neuropsychological Rehabilitation, University of Health and Rehabilitation Sciences Qingdao China; 9 School of Psychology, South China Normal University Guangzhou China; 10 Faculty of Health and Wellness, City University of Macau Macau China; 11 Beijing Key Laboratory of Applied Experimental Psychology, National Demonstration Center for Experimental Psychology Education (BNU), Faculty of Psychology, Beijing Normal University Beijing China; University of Toronto Canada; University College London United Kingdom

**Keywords:** computational psychiatry, mood fluctuations, depression, anxiety, reward prediction error, Human

## Abstract

Mood fluctuations, central to human experience, are profoundly influenced by reward prediction errors (RPE). Although depression and anxiety traditionally exhibit contrasting mood fluctuations, their interrelated nature has made it challenging to pinpoint their specific roles in RPE-induced mood variations. In this study, we employed a computational model of momentary mood within a gambling task, involving 2043 participants across five experiments. Participants also completed a battery of questionnaires designed to allow us to dissociate anxiety- and depression-specific traits through bifactor modeling. Results showed that depression was associated with dampened mood fluctuations due to mood hyposensitivity to RPE. Importantly, this pattern was also found in patients with affective disorders. In contrast, anxiety correlated with heightened mood fluctuations stemming from mood hypersensitivity to RPE in non-clinical participants. Moreover, the shared depression/anxiety component was linked to lower affective baseline and greater risk aversion. Collectively, our results uncover computational dissociation of depression vs. anxiety using RPE-based mood modeling and present multi-dimensional computational signatures for these symptoms, with clinical relevance for management of mood disorders.

## Introduction

Happiness is a central component of human experience, providing a subjective signal of value and guiding actions aimed at maximizing well-being (Bentham, 1780; Mill, 1859). Yet, well-being is often disrupted, as evidenced by the high prevalence of mood disorders such as depression and anxiety. It is therefore crucial to understand what drives mood fluctuations, and how these processes promote happiness or contribute to mood disorders.

Mood dynamics are strongly influenced by reward prediction error (RPE) – discrepancies between expected and actual outcomes (Eldar and Niv, 2015; Kao et al., 2023; Emanuel and Eldar, 2023; Bennett et al., 2022; Eldar et al., 2016; Rutledge et al., 2014; Otto et al., 2016). This influence is particularly prominent in uncertain environments, such as gambling scenarios, where outcomes frequently deviate from expectations and thereby drive moment-to-moment mood changes (Rutledge et al., 2014; Rutledge et al., 2015; Rutledge et al., 2016a; Blain and Rutledge, 2020; Vanhasbroeck et al., 2021). Although RPE-induced mood fluctuations play a critical role in adaptive behavior (Kao et al., 2023; Eldar et al., 2016; Quoidbach et al., 2019; Taquet et al., 2016), atypical mood dynamics may increase vulnerability to affective disorders (Kao et al., 2023; Broome et al., 2015; Mason et al., 2017; Taquet et al., 2020). Specifically, depression, often associated with blunted emotional responses (Bylsma et al., 2008; Rottenberg et al., 2005), may be linked to dampened mood fluctuations (Rottenberg et al., 2002a; Rottenberg et al., 2002b; Rottenberg and Hindash, 2015; Koval et al., 2012; Rutledge et al., 2017). In contrast, anxiety, characterized by exaggerated responses to uncertainty (Grupe and Nitschke, 2013), might intensify them (Bowen et al., 2006; Bowen et al., 2004). However, distinguishing the unique effects of depression and anxiety on mood dynamics presents a significant challenge, owing to their overlapping symptoms and entangled nature (Stavrakaki and Vargo, 1986; Steer et al., 1995).

Recent work has used bifactor models of the tripartite model of depression and anxiety to clarify their distinct features and differential influences on decision-making (Gagne et al., 2022; Gagne et al., 2020). The tripartite model of anxiety and depression proposes that these two symptom dimensions share a broad general distress or negative affect component while also including symptom-specific components: low positive affect/anhedonia is more specific to depression, whereas physiological hyperarousal is more specific to anxiety (Steer et al., 1995; Simms et al., 2008; Clark and Watson, 1991). Bifactor analysis offers a way to model this structure statistically. In a bifactor model, symptoms load on a general factor reflecting their shared variance and on specific factors capturing residual variance in narrower symptom dimensions after accounting for the general factor. Although bifactor and hierarchical models have long been used in psychometrics, for example intelligence research (Rodriguez et al., 2016; Reise, 2012), their application to anxiety and depression is grounded in the tripartite model and subsequent psychometric work distinguishing general internalizing/distress from symptom-specific dimensions. This framework has recently been extended to computational psychiatry, where shared and specific affective symptom dimensions have been linked to task-derived computational parameters. For example, Gagne et al., 2022 used bifactor analysis to show that depression was associated with weaker prior beliefs, whereas anxiety was associated with a stronger negative bias in belief updating (Gagne et al., 2022). Intriguingly, mood sensitivity to RPE appears to remain intact in both patients with major depressive disorder (MDD) and individuals with high depression scores (Blain and Rutledge, 2020; Rutledge et al., 2017; Csukly et al., 2023), contrary to the hypothesis that depression is associated with reduced RPE-related mood sensitivity (Rutledge et al., 2017). Individuals with anxiety not only show heightened vigilance before an outcome is known but may also assign greater precision to information that resolves uncertainty (Tobias and Ito, 2021; Huang et al., 2017). Such increased precision-weighting could amplify the affective influence of RPEs, leading to larger mood shifts when outcomes deviate from expectations (Villano et al., 2023; Paulus and Yu, 2012). Given evidence suggesting opposite associations of depression and anxiety with mood fluctuations (Rottenberg and Hindash, 2015; Koval et al., 2012; Bowen et al., 2006; Bowen et al., 2004), one possible explanation for the apparent intactness of RPE-related mood sensitivity (Rutledge et al., 2017) is that depression and anxiety exert opposing effects on this sensitivity, thereby counteracting each other. To test this hypothesis, we applied a bifactor model to disentangle these interrelated influences.

This study used a computational model of momentary mood in a gambling task across five experiments involving 2043 participants. Participants also completed a series of questionnaires, enabling bifactor analysis to dissociate the influences of anxiety- and depression-specific traits on mood fluctuations. We measured momentary mood by asking participants, “How happy are you at this moment?” This measure has also been used to index happiness or momentary subjective well-being (Rutledge et al., 2014; Quoidbach et al., 2019; Taquet et al., 2016; Jangraw et al., 2023). Although mood can persist for hours, days, or even weeks (Kao et al., 2023; Emanuel and Eldar, 2023; Eldar et al., 2016; Schiller et al., 2024), momentary mood measured in laboratory settings captures how multiple events accumulate to shape affective state over minutes (Kao et al., 2023; Eldar et al., 2016; Rutledge et al., 2014; Rutledge et al., 2016a; Blain and Rutledge, 2020; Vanhasbroeck et al., 2021; Rutledge et al., 2017; Csukly et al., 2023; Vinckier et al., 2018). Its external validity is supported by associations with depressive symptoms (Blain and Rutledge, 2020; Rutledge et al., 2017). We first validated the tripartite model of depression and anxiety before examining the specific roles of depression and anxiety in mood fluctuations (n=901). Next, we measured mood sensitivity to RPE in a gambling task and investigated its relation to anxiety- and depression-specific traits based on the tripartite model in a laboratory experiment (n=44) and two additional online experiments (n=747 and n=235). In the final experiment (n=116), we tested the generalizability of these findings in a clinical sample of patients with affective disorders, revealing dissociable roles of depression and anxiety in RPE-related mood dynamics.

## Results

### Experimental protocol

**Figure 1.**
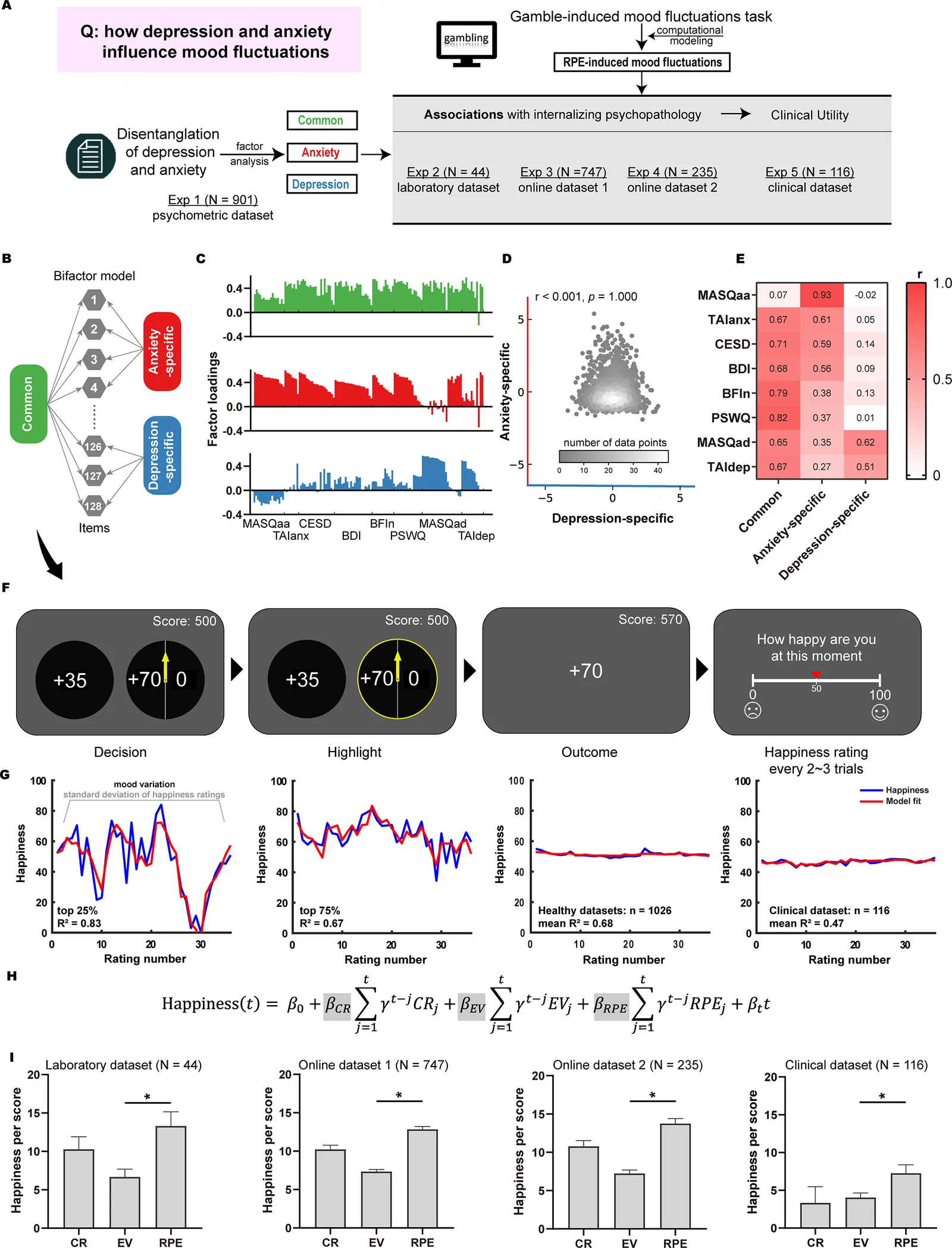
Experimental protocol. (**A**) Study outline. This study examines how depression and anxiety influence mood fluctuations. The first experiment assesses the bifactor structure that disentangles shared and specific components of depression and anxiety in the psychometric dataset (N=901). The second experiment tests associations between depression- and anxiety-specific traits and RPE-induced mood fluctuations using the questionnaire battery and a gambling task with momentary mood ratings in the laboratory dataset (N=44). The third and fourth experiments replicate the second experiment in online samples (online dataset 1, N=747; online dataset 2, N=235). The fifth experiment tests whether these findings generalize to a clinical dataset of patients with affective disorders (N=61). (**B**) Mapping between the three factors and 128 items in the bifactor model. (**C**) Factor loadings of items on the general factor, anxiety-specific factor, and depression-specific factor. (**D**) Orthogonality among the general, anxiety-specific, and depression-specific factor scores across datasets with complete questionnaire data. (**E**) Mean correlations between factor scores and questionnaire scores. Overall, the general factor showed high correlations with all questionnaires. The depression-specific factor correlated most strongly with TAIdep and MASQad, whereas the anxiety-specific factor correlated most strongly with the remaining questionnaires. These correlational results supported the bifactor structure of anxiety and depression. (**F**) Gambling task design. On each trial, participants were asked to choose between a certain option and a gamble option. Once an option was selected, the corresponding outcome was displayed in the center of the screen. The cumulative score was always shown in the upper-right corner. Every two or three trials, participants were asked to complete a self-paced rating of “How happy are you at this moment?” on a slider scale ranging from 0 (very unhappy) to 100 (very happy). (**G**) Temporal dynamics of happiness ratings for representative individuals with high (top 25%) and low (top 75%) mood variation, healthy datasets, and the clinical dataset. (**H**) Momentary mood model. (**I**) Results of momentary mood model for each dataset. MASQaa, the subscale of anxious arousal in the Mood and Anxiety Symptoms Questionnaire; TAIanx, the subscale of anxiety in the Trait Anxiety Inventory; CESD, Center for Epidemiologic Studies Depression Scale; BDI, Beck Depression Inventory; BFIn, the subscale of neuroticism in the Big Five Inventory; PSWQ, Penn State Worry Questionnaire; MASQad, the subscale of anhedonic depression in the Mood and Anxiety Symptoms Questionnaire; TAIdep, the subscale of depression in the Trait Anxiety Inventory; CR, certain reward; EV, expected value; RPE, reward prediction error.

After completing a battery of questionnaires (Gagne et al., 2020; see Materials and methods), participants performed a gambling task with momentary mood ratings (Rutledge et al., 2014; Rutledge et al., 2015; Vanhasbroeck et al., 2021). Within this task, participants were asked to choose between a certain option and a gamble option with two possible outcomes, each occurring with a 50% probability. Participants were instructed to rate their mood every two to three trials (Figure 1). Detailed participant demographics are summarized in Table 1. Choice data (e.g. gambling rates) and mood data (e.g. initial mood, mean mood, and mood variation) showed patterns similar to those reported in previous studies measuring momentary mood during gambling tasks (Figure 1—figure supplements 1 and 2; Rutledge et al., 2015; Rutledge et al., 2016b). We also replicated established effects on momentary mood: mood was higher following gains than following losses, and mood drifted over time (all p*s* <0.001; Figure 1—figure supplement 3).

**Figure 1—figure supplement 1.**
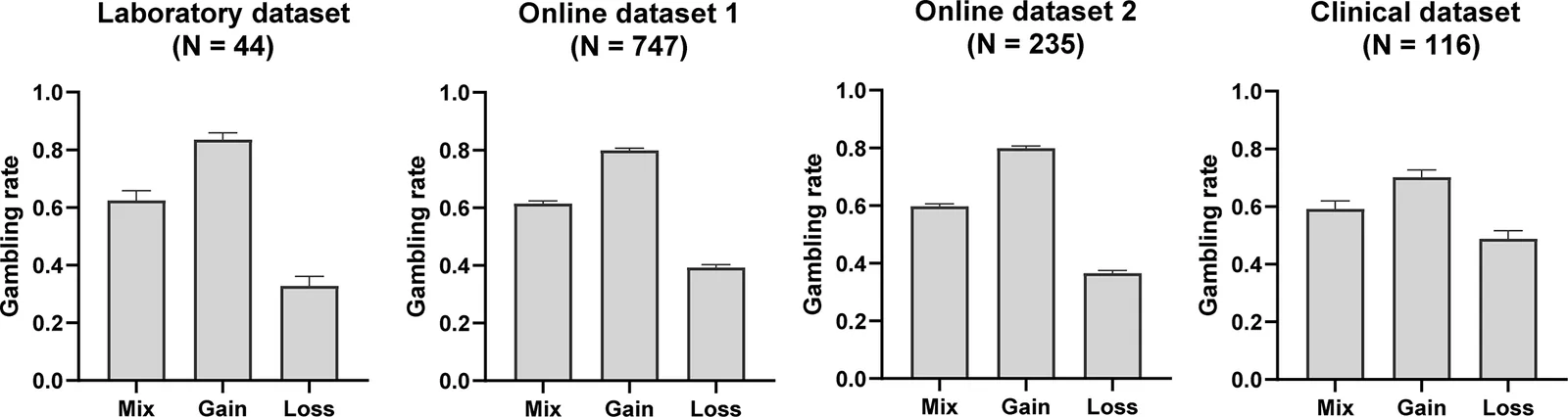
Gambling rate in each dataset. All datasets showed the same gambling rate pattern, as well as in line with literature.

**Figure 1—figure supplement 2.**
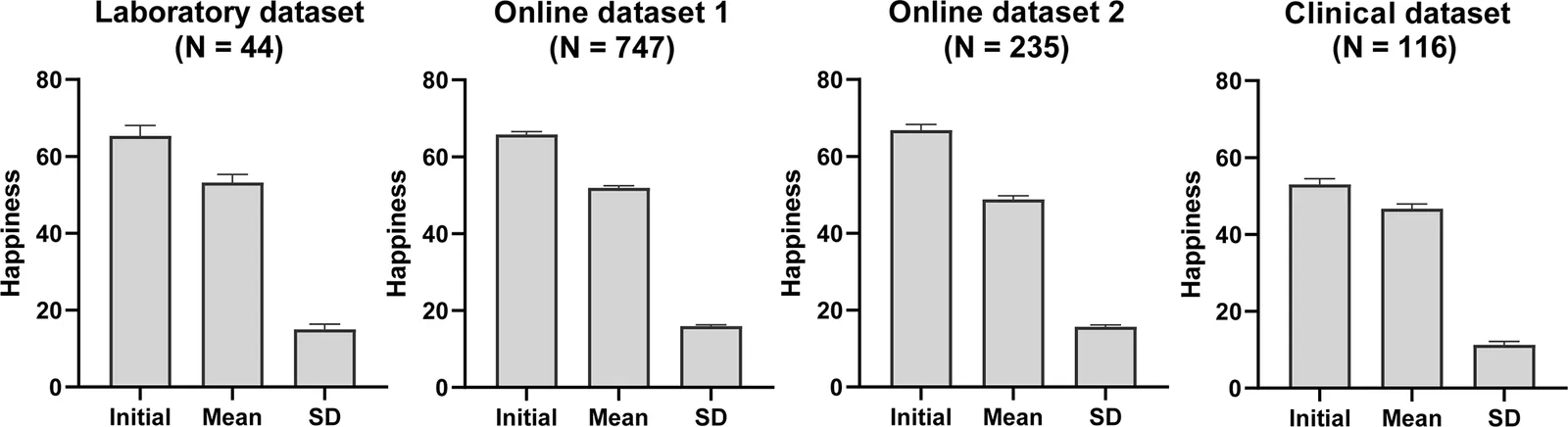
Model-agnostic mood results for all datasets. All datasets showed the same initial mood overall before the task, mean mood, and mood variation across the task.

**Figure 1—figure supplement 3.**
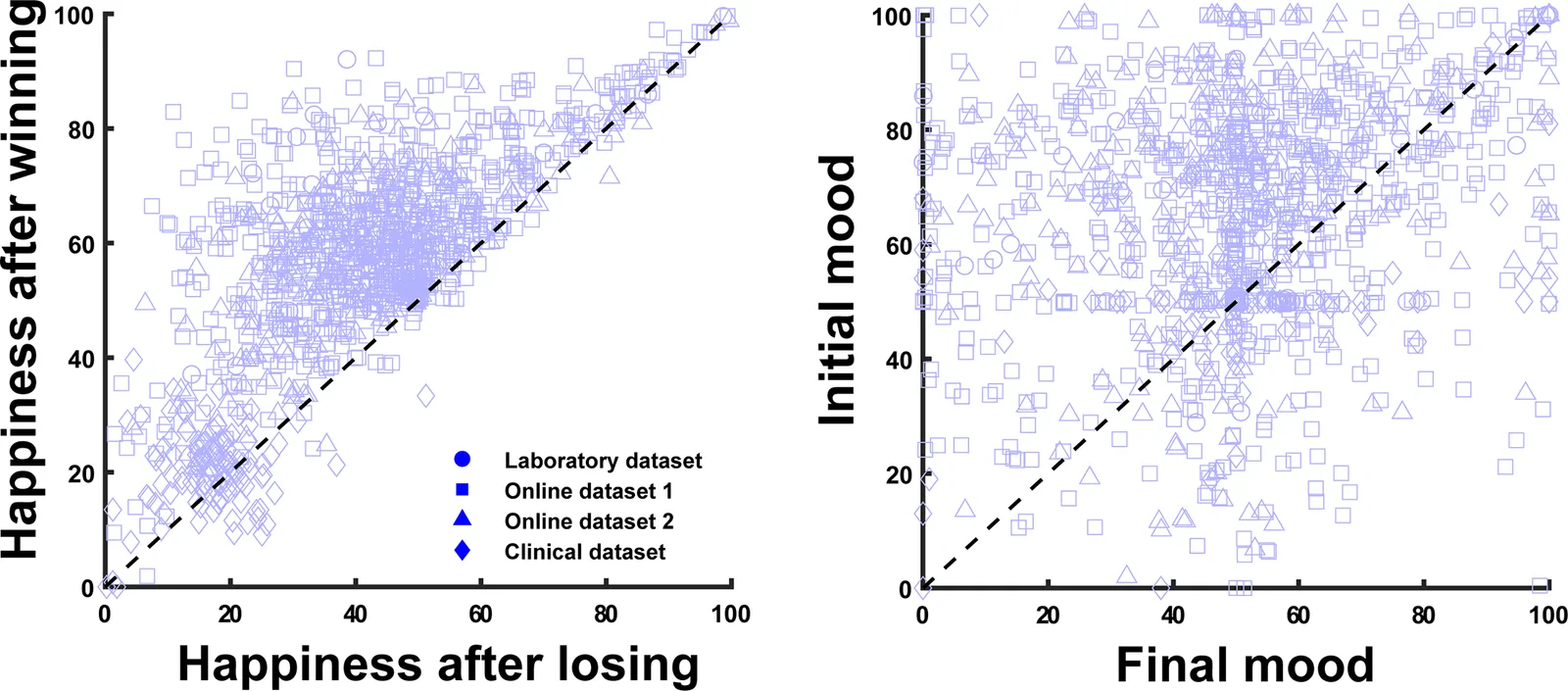
Replication of the classic mood effects. (**A**) Happier after winning than losing. (**B**) Mood drift across the task.

**Figure 1—figure supplement 4.**
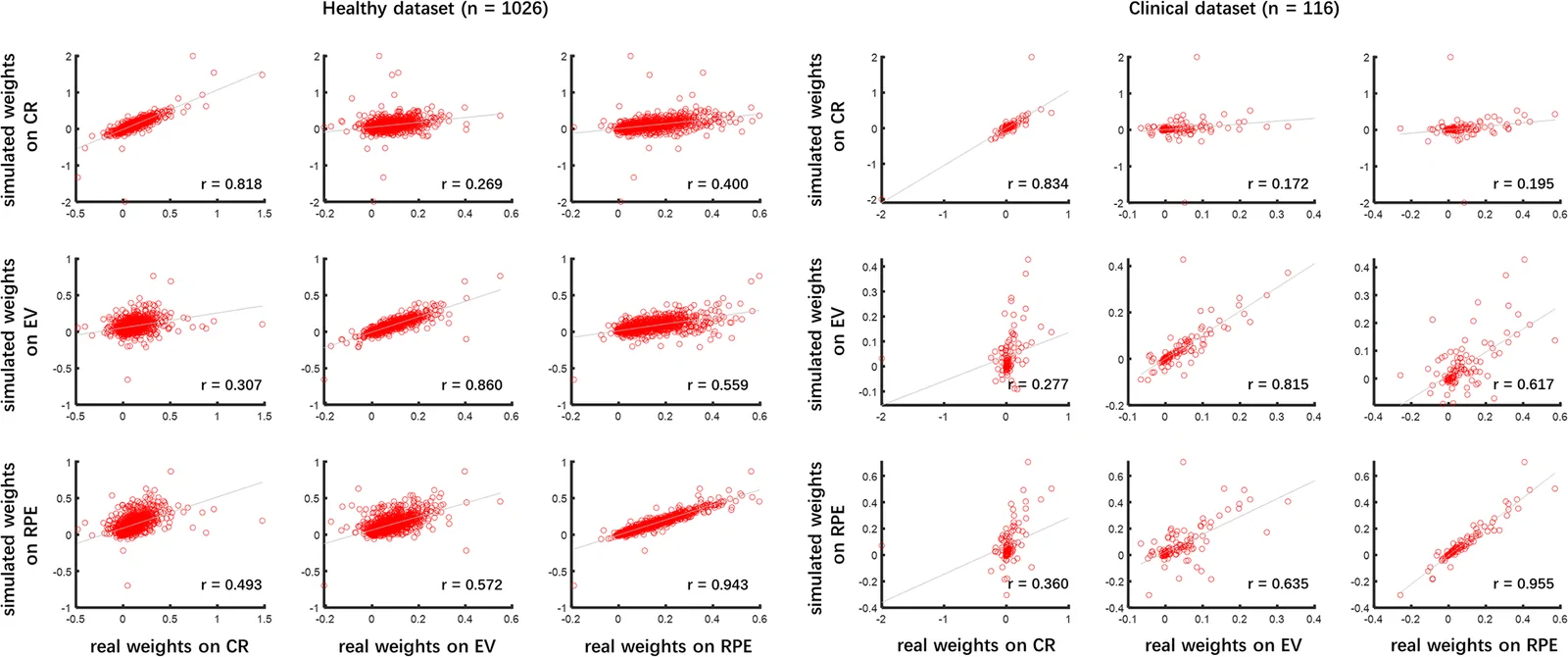
Parameter recovery for the winning model (M1) for the healthy and clinical datasets. Parameter recovery analysis showed high correlations between the matched simulated and real parameters and low correlations between the mismatched simulated and real parameters.

**Figure 1—figure supplement 5.**
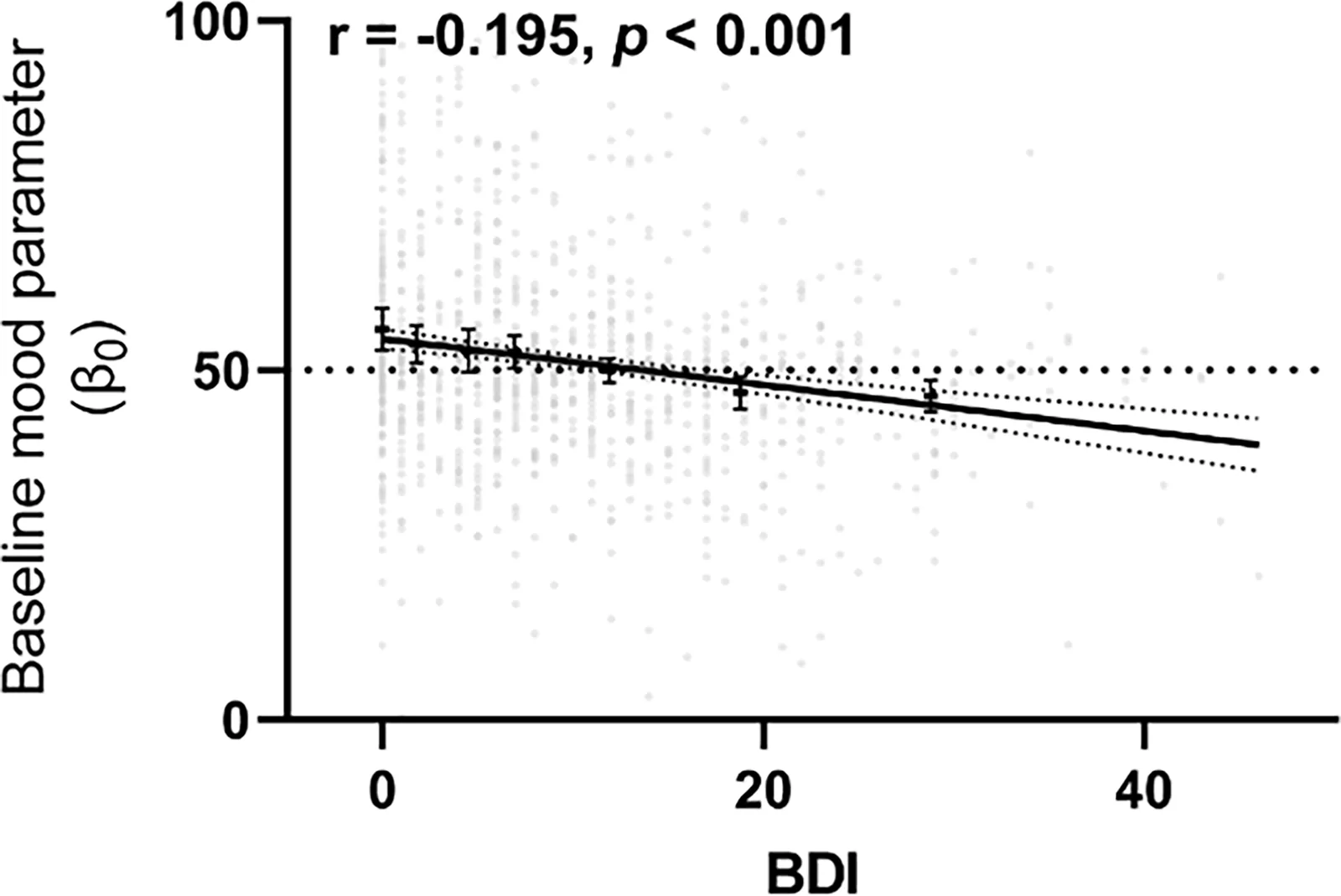
Replication of Rutledge et al., 2017’s findings using BDI (N=1026). Depression symptom measured by BDI was negatively correlated with the baseline mood parameter. Data was combined from laboratory dataset, online dataset 1, and online dataset 2.

**Figure 1—figure supplement 6.**
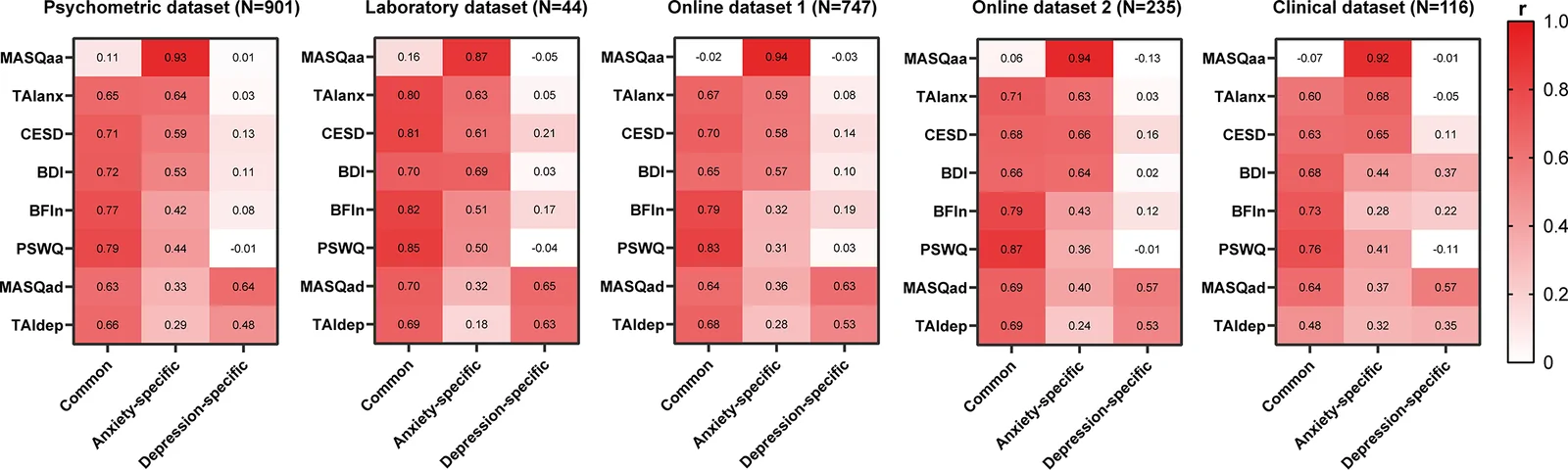
Correlations of factor scores from the winning model (the bifactor model) with questionnaire scores for each dataset.

**Table 1.** Basic demographic details.

	Psychometric dataset (N=901)	Laboratory dataset (N=44)	Online dataset 1 (N=747)	Online dataset 2 (N=235)	Clinical dataset (N=116)
Gender (female)	676	18	500	143	89
Age	22.04±2.10	20.05±1.70	20.90±2.41	21.67±2.52	16.40±3.86
MASQaa	24.70±8.13	22.98±6.14	24.16±7.60	23.92±7.62	43.51±10.51
TAIanx	16.06±4.86	15.00±4.95	15.69±4.97	15.99±5.34	23.35±5.44
CESD	35.28±10.72	33.30±10.73	34.82±10.74	35.06±11.41	55.64±13.04
BDI	11.38±9.35	7.91±8.11	10.04±8.94	10.26±9.18	22.66±17.91
BFIn	33.99±8.82	30.52±8.26	32.82±9.37	32.79±9.81	43.75±9.31
PSWQ	48.28±11.61	46.89±12.52	48.25±12.15	48.36±13.29	59.18±12.87
MASQad	61.49±14.00	60.23±16.63	58.07±15.30	59.57±15.71	77.16±15.39
TAIdep	29.39±5.47	27.32±6.16	27.61±6.27	27.85±6.34	35.97±4.91
Data collection periods	2021.6.22–2021.6.28	2022.6.2–2022.7.5	2022.4.24–2022.5.22	2022.7.22–2022.7.26	2023.3.16–2023.8.4

Descriptive data are presented as mean ± SD.

MASQaa, the subscale of anxious arousal in the Mood and Anxiety Symptoms Questionnaire; TAIanx, the subscale of anxiety in the Trait Anxiety Inventory; CESD, Center for Epidemiologic Studies Depression Scale; BDI, Beck Depression Inventory; BFIn, the subscale of neuroticism in the Big Five Inventory; PSWQ, Penn State Worry Questionnaire; MASQad, the subscale of anhedonic depression in the Mood and Anxiety Symptoms Questionnaire; TAIdep, the subscale of depression in the Trait Anxiety Inventory.

To examine the computational drivers of momentary mood, we used the classic mood model, assuming that momentary mood is modeled as a recency-weighted sum of certain rewards from chosen certain options (CR), expected values of chosen gambles (EV), and RPEs following gamble outcomes (Equation 1; Figure 1H; Rutledge et al., 2014; Vanhasbroeck et al., 2021). RPE was defined as the difference between the obtained outcome and the expected value of the chosen gamble. We also incorporated a drift parameter to account for gradual changes in happiness over time (Jangraw et al., 2023; Vinckier et al., 2018).

(1) $$\displaystyle  {\rm Happiness}\left (t\right)=\beta _{0}+\beta _{CR}\sum _{j=1}^{t}\gamma ^{t-j}CR_{j}+\beta _{EV}\sum _{j=1}^{t}\gamma ^{t-j}EV_{j}+\beta _{RPE}\sum _{j=1}^{t}\gamma ^{t-j}RPE_{j}+\beta _{t}t$$

This model explained momentary moods well across all gambling-task datasets (R^2^: mean ± SD = 0.67±0.20 for the laboratory dataset, 0.69±0.17 for online dataset 1, and 0.64±0.19 for online dataset 2, and 0.47±0.21 for the clinical dataset; see Appendix 2 and Note 5 for mood model space; see Appendix 1—tables 2 and 4 for mood model comparisons), with good performance for parameter recovery (Figure 1—figure supplement 4). Across all gambling-task datasets, we replicated previous model-based findings on momentary mood (Rutledge et al., 2014; Vanhasbroeck et al., 2021): (1) the RPE weight was significantly higher than the EV weight (ts >3.84, p*s* <0.001; Figure 1I), suggesting that momentary mood was more strongly influenced by RPEs following gamble outcomes than by the expected value of chosen gamble; (2) the baseline mood parameter $\beta _{0}$ was positively correlated with the initial mood ratings (rs >0.25, p*s* <0.006), supporting the interpretability of this model parameter. We also replicated previous depression-related findings (Rutledge et al., 2017): depression symptom measured by Beck Depression Inventory (BDI) was negatively correlated with the baseline mood parameter $\beta _{0}$ (*r*=–0.173, p<0.001; Figure 1—figure supplement 5). In sum, these confirmatory results support the validity of the questionnaire and task measures, the substantial contribution of RPEs to momentary mood fluctuations, and a robust association between BDI-measured depressive symptoms and the baseline mood parameter.

### Depression is associated with dampened mood fluctuations through reduced sensitivity to RPEs

We first examined the association between depression and mood fluctuations, as well as its computational signature. Depression and anxiety often co-occur at the symptom level (Stavrakaki and Vargo, 1986; Clark and Watson, 1991; Briley et al., 2022; Wang et al., 2021), as reflected by a high correlation between depressive symptoms measured by the BDI and anxiety symptoms measured by the anxiety subscale of the Trait Anxiety Inventory (*r*=0.75, p<0.001). To differentiate the unique influences of anxiety- and depression-specific factors, we applied the validated bifactor structure from the psychometric dataset (Figure 1C) to decompose depression and anxiety symptoms into three orthogonal components: the general factor, anxiety-specific factor, and depression-specific factor (–0.001<*rs* < 0.001, all p*s* = 1.000; see Appendix 1 for psychometric properties of the tripartite model of depression and anxiety in our sample; n=901; Figure 1—figure supplement 6).

**Figure 2.**
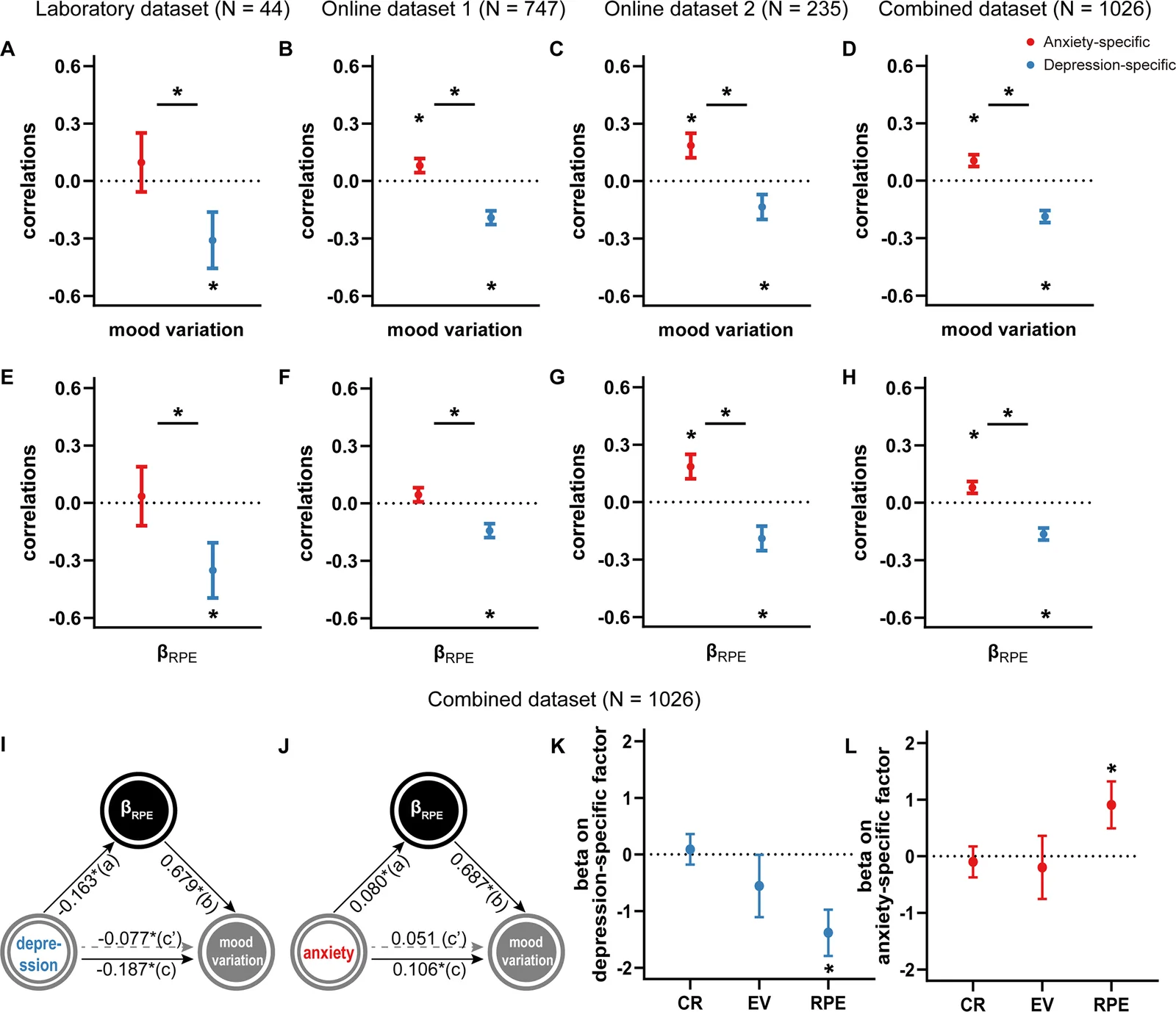
Dissociable associations of depression and anxiety with mood fluctuations. Correlations of depression- and anxiety-specific factor scores with mood variation and RPE-related mood sensitivity ($\beta _{RPE}$) for the laboratory dataset (**A, E**), online dataset 1 (**B, F**), online dataset 2 (**C, G**), and the combined dataset (N=1026; **D, H**). (**I**) Reduced RPE-related mood sensitivity statistically mediated the association between depression-specific scores and reduced mood fluctuations. (**J**) Increased RPE-related mood sensitivity statistically mediated the association between anxiety-specific scores and increased mood fluctuations. (**K**) Among the mood sensitivity parameters, the depression-specific factor was selectively associated with decreased RPE-related mood sensitivity. (**L**) Among the mood sensitivity parameters, the anxiety-specific factor was selectively associated with increased RPE-related mood sensitivity. Regression coefficients are shown as bootstrapped mean ± SE. CR, certain reward; EV, expected value; RPE, reward prediction error. *p<0.05.

To examine whether depression-specific factor scores were associated with mood fluctuations, we performed correlation analyses between the depression-specific factor and mood variation, defined as the SD of happiness ratings across trials. We found convergent results across the laboratory dataset, online dataset 1, and online dataset 2. Specifically, depression-specific scores were negatively correlated with mood variation (rs <–0.13, p*s* <0.041; Figure 2A–C). Model-based correlational analyses further showed negative correlations between depression-specific scores and RPE-related mood sensitivity ($\beta _{RPE}$; *rs* <–0.14, p*s* <0.019; Figure 2E–G), which remained significant after controlling for gender, age, task earnings, and mood drift (p*s* <0.035). Bootstrap validation yielded consistent results, indicating that higher depression-specific scores were associated with reduced mood fluctuations and decreased RPE-related mood sensitivity. Given the strong correlation between β_RPE_ and mood variation (*r*s >0.67, ps <0.001), we further conducted a mediation analysis to examine whether individual differences in RPE-related mood sensitivity statistically accounted for the association between depression loading and mood variation. This analysis was motivated by the hypothesis that depression-related dampening of mood variability may arise, at least in part, from reduced mood sensitivity to RPEs. The results supported our hypothesis (p*s* <0.015; Figure 2I), suggesting that higher depression-specific scores are associated with dampened momentary mood variation through reduced RPE-related mood sensitivity. See Appendix 1—table 7 for full statistical results for each dataset. To further test whether the association between depression and mood fluctuations was specific to RPE-related mood sensitivity, we regressed depression-specific factor scores on mood sensitivities to CR, EV, and RPE in the combined dataset (N=1026). Only RPE-related mood sensitivity was significantly associated with depression-specific scores (CR: *t*=0.274, p=0.784; EV: *t*=–1.069, p=0.285; RPE: *t*=–3.282, p=0.001; Figure 2K).

### Anxiety is associated with intensified mood fluctuations through increased sensitivity to RPEs

We then examined how anxiety was associated with mood fluctuations. Correlations between the anxiety-specific factor and mood variation were positive in direction across datasets, although they were not statistically significant in several datasets (the laboratory dataset: *r*=0.10, p=0.531; the online dataset 1: *r*=0.08, p=0.026; the online dataset 2: *r*=0.19, p=0.004). Similarly, correlations between the anxiety-specific factor and $\beta _{RPE}$ were positive in direction but statistically inconsistent across datasets (the laboratory dataset: *r*=0.04, p=0.820; the online dataset 1: *r*=0.05, p=0.216; the online dataset 2: *r*=0.19, p=0.004; Figure 2A–C & and E–G). Because these datasets used comparable task and questionnaire procedures and showed positive effect directions, and because reliable individual differences often require large samples to detect (Marek et al., 2022), we combined the laboratory dataset, online dataset 1, and online dataset 2 (total N=1026). This approach is analogous to an individual-participant-data meta-analytic analysis. We fitted linear mixed-effects models predicting mood variation and $\beta _{RPE}{\ }$ from the three bifactor scores, with dataset included as a random intercept to account for dataset-level variability. For mood variation, the anxiety-specific factor was positively associated with mood variation (*t*=3.46, p<0.001), whereas the depression-specific factor was negatively associated with mood variation (*t*=–6.13, p<0.001). For RPE-related mood sensitivity, the anxiety-specific factor was positively associated with $\beta _{RPE}$ (*t*=2.60, p=0.009), whereas the depression-specific factor was negatively associated with $\beta _{RPE}$ (*t*=–5.30, p<0.001). These associations remained significant after controlling for gender, age, task earnings, and mood drift. In addition, we performed a mini meta-analysis on these correlation coefficients (Goh et al., 2016). Results showed significant positive correlation for both mood variation and RPE-related mood sensitivity (mood variation: Z=3.399, 95 % CI for correlation coefficient *r* [0.045, 0.166]; RPE-related mood sensitivity: Z=2.618, 95 % CI for correlation coefficient *r* [0.021, 0.143]), supporting that anxiety is associated with intensified mood fluctuations and increased mood sensitivity to RPE in non-clinical participants. Mediation analysis further showed that heightened mood sensitivity to RPEs statistically mediated the association between anxiety loading and greater mood fluctuations (*a×b* = 0.055, 95% CI = [0.013, 0.097], p=0.010; Figure 2J). Moreover, linear regression against anxiety-specific factor with mood sensitivity to CR, EV, and RPE as regressors showed RPE-specific mood hyper-sensitivity with anxiety loading (CR: t=–0.36, p=0.718; EV: t=–0.35, p=0.724; RPE: t=2.19, p=0.028; Figure 2L).

To address the possibility that individual differences in subjective scale calibration confounded our findings, we conducted several complementary analyses. For example, individuals with nonlinear utility functions, such as risk-seeking participants, might show disproportionately large mood responses to large versus small wins. We therefore re-analyzed two publicly available datasets using similar risky decision-making tasks with repeated happiness ratings: (n=49; Vanhasbroeck et al., 2021) and the Rutledge smartphone app dataset (n=46,204). See Appendix 3 for details. Collectively, these analyses suggested that individual differences in risk preference did not account for our primary findings regarding distinct mood dynamics in anxiety versus depression. These results further suggest a partial dissociation between decision-related risk preferences and RPE-related mood dynamics. Together, these results suggest that depression and anxiety are associated with opposite patterns of mood dynamics through RPE-specific mood sensitivity.

### Differential associations of depression and anxiety with mood fluctuations

To directly test whether depression- and anxiety-specific factors differed in their associations with mood dynamics, we compared the corresponding correlations. These comparisons showed that depression-specific associations were significantly more negative than anxiety-specific associations for both mood variation (laboratory dataset: *Z*=–1.84, p=0.033; online dataset 1: *Z*=–5.36, p<0.001; online dataset 2: *Z*=–3.42, p<0.001) and $\beta _{RPE}$ (laboratory dataset: *Z*=–1.77, p=0.038; online dataset 1: *Z*=–3.67, p<0.001; online dataset 2: *Z*=–4.00, p<0.001; Figure 2A–C , and E–G). These results support distinct associations of depression- and anxiety-specific factors with RPE-related mood dynamics.

### Clinical relevance of reduced RPE-related mood fluctuations in depression

**Figure 3.**
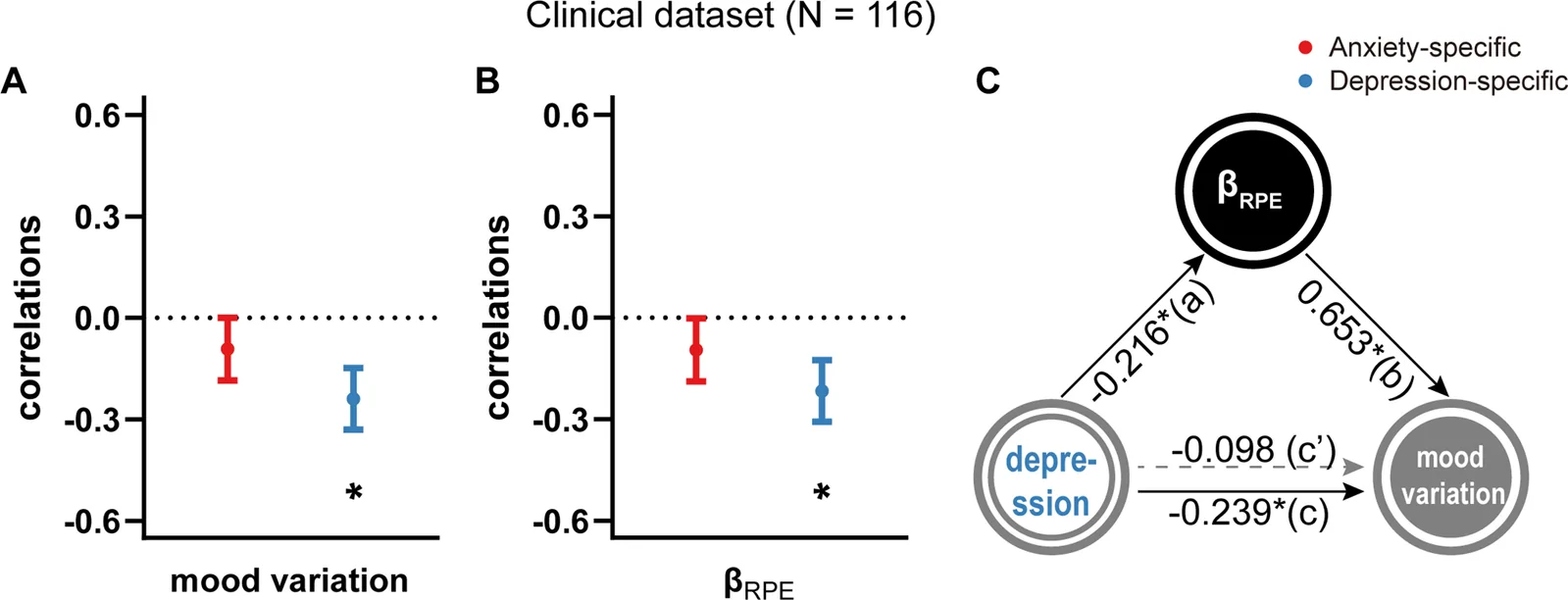
Clinical validation of reduced RPE-related mood sensitivity in depression. (**A**, **B**) Correlations of depression and anxiety factor score with mood variation and mood parameter of RPE ($\beta _{RPE}$) for the clinical dataset. (**C**) The mediation model among depression, $\beta _{RPE}$, and mood variation in the clinical population. The regression coefficients were represented by mean ± SE, which were estimated by bootstrap. RPE, reward prediction error; *p<0.05.

To test whether abnormalities in RPE-driven mood fluctuations can serve as clinically relevant computational markers of depression- and anxiety-related symptom dimensions, we recruited patients with affective disorders (n=116) to complete the same questionnaire battery and gambling task with momentary mood ratings (Figure 1). Demographic, psychological, and clinical characteristics are summarized in Table 1, Appendix 1—table 8. We observed significant negative correlations between depression-specific scores and both mood variation (*r*=–0.239, p=0.009) and RPE-related mood sensitivity ($\beta _{RPE}$; *r*=–0.216, p=0.020). These associations remained significant after controlling for demographic and clinical covariates, task earnings, and mood drift (p*s* <0.05). Bootstrap validation yielded consistent results. Mediation analyses further showed that reduced mood sensitivity to RPEs statistically mediated the association between depression-specific scores and lower mood fluctuations (a×b = –0.141, 95% CI = [-0.261,–0.038], p=0.021; Figure 3). However, we did not observe significant correlation with anxiety (mood variation: *r*=–0.092, p=0.327; $\beta _{RPE}$: *r*=–0.095, p=0.311).

### Exploratory associations of the common factor with mood and choice parameters

Given the theoretical relevance of the common factor – often linked to shared symptoms such as somatic complaints, sleep disturbances, and cognitive impairments (Clark and Watson, 1991) – as well as the potential for nonlinear associations with symptom severity, we conducted exploratory analyses to examine its relationship with choice and mood parameters in both healthy and clinical datasets. Choice parameters were estimated using an established approach–avoidance prospect theory model (Rutledge et al., 2015; Rutledge et al., 2016b; Wang et al., 2026), which included loss aversion, domain-specific risk attitude parameters in the gain and loss domains, and value-independent Pavlovian approach and avoidance parameters (see Appendix 7 for details of the computational choice models). In this model, risk attitude was quantified by the exponent parameter α in a prospect-theory-inspired subjective value function. Lower α values reflect greater risk aversion, whereas values closer to or above 1 reflect more linear or risk-seeking valuation.

**Figure 4.**
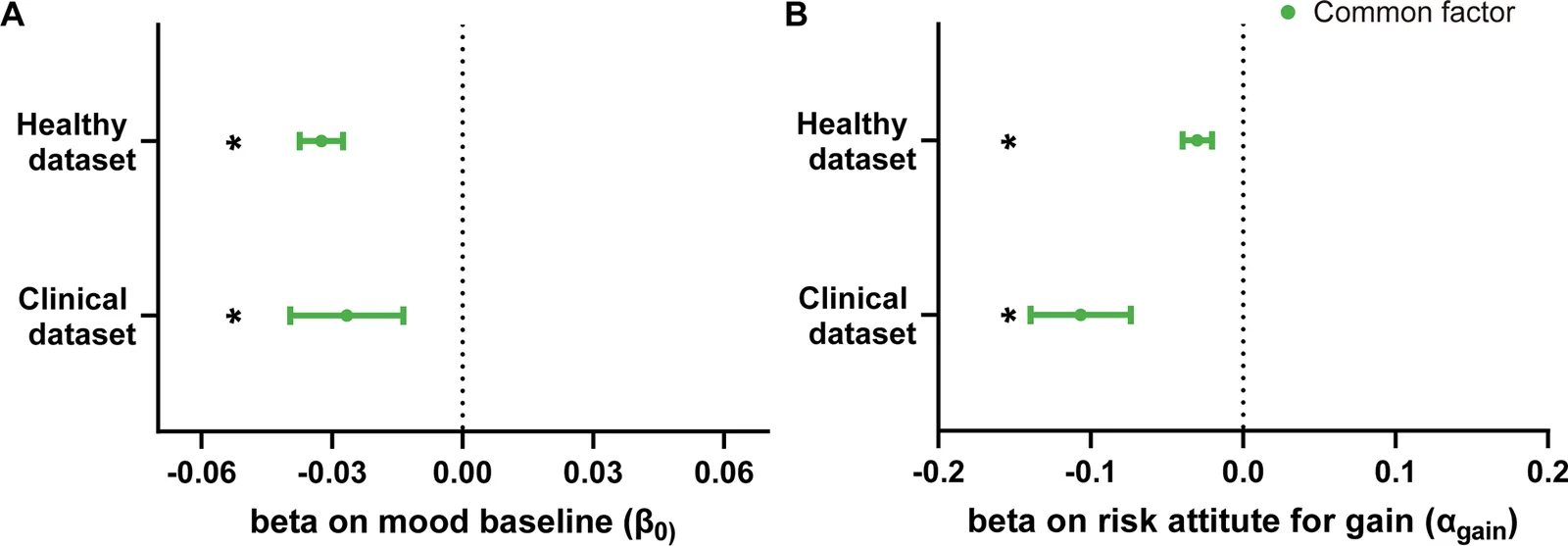
Consistent negative associations of the common factor with mood baseline and gain-domain risk attitude across datasets. (**A**) Negative association between the common factor and the baseline mood parameter. (**B**) Negative association between the common factor and gain-domain risk attitude. Regression coefficients are shown as mean ± SE. *p<0.05.

To improve robustness and reduce the risk of false positives, we focused on findings that were consistent across datasets. Model comparisons using BIC consistently favored linear over nonlinear models for associations between the common factor and mood and choice parameters (Table 2). Specifically, we observed significant negative associations between the common factor and two key variables: the baseline mood parameter and gain-domain risk attitude (Figure 4). Notably, these parameters were not consistently related to either the depression-specific or anxiety-specific factor across healthy and clinical datasets (depression ×baseline mood parameter: *r*=–0.12, p<0.001 for the healthy datasets and *r*=–0.08, p=0.934 for the clinical dataset; anxiety ×baseline mood parameter: *r*=–0.14, p<0.001 for the healthy datasets and *r*=–0.15, p=0.104 for the clinical dataset; depression ×risk attitude for gain: *r*=–0.02, p=0.505 for the healthy datasets and *r*=–0.00, p=0.961 for the clinical dataset; anxiety ×risk attitude for gain: *r*=0.01, p=0.846 for the healthy datasets and *r*=0.04, p=0.688 for the clinical dataset). These findings suggest that higher common-factor scores, reflecting general internalizing psychopathology, are associated with a lower affective baseline and greater gain-domain risk aversion, consistent with the idea that the common factor captures transdiagnostic features shared across mood and anxiety disorders.

**Table 2.** Exploratory analysis for the common factor.

Models	Healthy datasets (n=1026)		Clinical dataset (n=116)	
	BIC	Results	BIC	Results
**β_0_** ~ common	**–830.23**	b=–0.03, t=–6.54, p<0.001, 95% CI = [-0.042–0.022]	**–113.74**	b=–0.03, t=–2.05, p=0.043, 95% CI = [-0.052–0.001]
**β_0_** ~ common + common^2^	–823.74		–110.91	
**α_gain_** ~ common	**551.76**	b=–0.03, t=–3.08, p=0.002, 95% CI = [-0.049–0.011]	**101.7**	b=–0.11, t=–3.24, p=0.002, 95% CI = [-0.172–0.041]
**α_gain_** ~ common + common^2^	553.73		106.12	

Note: For healthy datasets, we added datasets as random variables. **β_0_**, the mood baseline parameter; **α_gain_**, risk attitude for gain.

## Discussion

Although atypical mood dynamics are considered central features of depression and anxiety (Rottenberg et al., 2002a; Rottenberg and Hindash, 2015; Bowen et al., 2006; Bowen et al., 2004), little is known about their underlying computational mechanisms. Our study offers computational insights into the distinct associations of depression and anxiety with mood fluctuations. By orthogonally decomposing shared and specific symptom variance in depression and anxiety, we were able to distinguish their specific associations with mood dynamics. Depression-specific symptoms were associated with dampened mood fluctuations through reduced mood sensitivity to RPEs, whereas anxiety-specific symptoms were linked to intensified mood fluctuations through increased mood sensitivity to RPEs in non-clinical datasets. Notably, the depression-related reduction in RPE-related mood sensitivity was replicated in patients with affective disorders, highlighting its potential clinical relevance as an affective computational marker. Moreover, the shared depression–anxiety component was linked to a lower affective baseline and greater risk aversion.

Depression and anxiety, though often coexisting (Stavrakaki and Vargo, 1986; Steer et al., 1995), show contrasting associations with mood dynamics, consistent with their distinct affective profiles (Koval et al., 2012; Bowen et al., 2006; Bowen et al., 2004). Our computational model not only replicates the important role of RPEs in mood dynamics but also highlights the divergent mediating roles of RPE-related mood sensitivity in the associations of depression and anxiety with mood fluctuations. The opposite associations of depression and anxiety with mood sensitivity to RPEs complement previous findings of apparently intact RPE-related mood sensitivity in depression (Blain and Rutledge, 2020; Rutledge et al., 2017; Csukly et al., 2023). These findings further underscore the necessity of decomposing shared and specific components of depression and anxiety in studies of mood dynamics, which can enhance our understanding of their distinct associations with emotion processing and cognitive flexibility. This point is consistent with bifactor-based work showing that shared and specific symptom dimensions can have different computational correlates. For example, Gagne et al., 2020 showed that bifactor-derived symptom dimensions differentially relate to maladaptation to environmental volatility (Gagne et al., 2020), complementing previous findings that trait anxiety is associated with inflexible adjustment to volatility (Gagne et al., 2020). Although the present study did not include neuroimaging, the observed computational dissociation may map onto partially distinct neural systems involved in reward learning, mood updating, and affective psychopathology. RPE processing has been consistently linked to striatal–midbrain dopaminergic reward-learning circuits (Rutledge et al., 2014; Schultz et al., 1997). The integration of these reward-learning signals into subjective mood and value-based decision-making may further involve the ventral medial prefrontal cortex and orbitofrontal cortex (Vinckier et al., 2018). In addition, the anterior insula may be particularly relevant for integrating feedback-related signals with affective and interoceptive states (Rutledge et al., 2014; Vinckier et al., 2018), potentially linking RPE processing to anxiety- and depression-related mood dynamics. Consistent with this view, Cecchi et al., 2022 used intracranial EEG to show that feedback-related neural activity tracks mood fluctuations and risky choice. Future neuroimaging studies should test whether depression-related reductions and anxiety-related increases in RPE-related mood sensitivity are associated with altered interactions among striatal, prefrontal, and insular circuits.

This study bridges research on affective dynamics and reward-based decision-making in depression. Anhedonia, a core symptom of depression, refers to a reduced ability to experience pleasure (Hall et al., 2024). In affective science, researchers have used experience-sampling methods over several weeks or months (Emanuel and Eldar, 2023; Eldar et al., 2016; Bowen et al., 2006; Pulcu et al., 2022), showing dampened mood fluctuations in depression (Rottenberg and Hindash, 2015; Koval et al., 2012). In parallel, decision-making and learning research has often adopted laboratory-based learning or gambling tasks, revealing alterations in reward processing in individuals with depression (Pike and Robinson, 2022; Bishop and Gagne, 2018). However, how altered reward processing translates into aberrant momentary hedonic experience remains largely unknown. Our study employed a gambling task with momentary mood ratings, allowing us to examine reward- and RPE-related mood dynamics. Consistent with both affective-science and reward-learning accounts (Rottenberg et al., 2005; Koval et al., 2012), this study provides evidence that depressive symptoms are associated with reduced mood sensitivity to RPEs.

In addition to supporting the proposed role of anxiety in intensified mood fluctuations (Grupe and Nitschke, 2013; Bowen et al., 2006; Bowen et al., 2004), our study provides a computational account of this phenomenon: anxiety was associated with heightened mood sensitivity to RPEs. Notably, the anxiety-related effects were less robust than the depression-related effects and were detectable only in the pooled dataset (n=1026); therefore, they require further replication in larger samples. Anxiety symptoms are often marked by exaggerated anticipatory responses to uncertainty and excessive affective reactivity (Grupe and Nitschke, 2013). Because RPEs quantify the discrepancy revealed when uncertain outcomes are resolved, our findings suggest a potential pathway through which anxiety may shape mood responses to uncertainty resolution – by amplifying affective responses to unexpected outcomes. In this way, individuals with higher anxiety may exhibit a stronger need to resolve uncertainty (Browning et al., 2015), which could contribute to increased mood reactivity to RPEs. Our findings differ from those of Browning et al., 2015, who reported reduced adaptation of learning rates to environmental volatility in anxiety. However, the two findings may reflect different aspects of uncertainty processing: while Browning et al., 2015 focused on learning-rate adaptation in response to environmental volatility, our study targets affective responsiveness to outcome-level prediction errors. Thus, anxious individuals may exhibit reduced cognitive flexibility in belief updating yet increased emotional reactivity to prediction errors, highlighting a possible dissociation between cognitive and affective systems. Notably, the pattern of heightened RPE sensitivity observed in the pooled non-clinical dataset was not observed in the clinical sample. On the one hand, this discontinuity may reflect that the clinical sample was underpowered to detect anxiety-specific effects, especially given the high comorbidity between anxiety and depression in affective disorders (Appendix 1—table 8). Based on the effect size observed in the non-clinical datasets (*r*=0.079), we estimated that a sample size of 1226 would be required to detect this effect with 80% statistical power using a two-tailed test with *α*=0.05. This estimate is substantially larger than the current clinical sample size (n=116). On the other hand, it may reflect a disruption of mood homeostasis in clinical populations (Paulus and Yu, 2012; Paulus, 2007). In non-clinical individuals, counterbalancing associations of depression- and anxiety-related traits with mood variation may help maintain emotional equilibrium. In contrast, affective disorders may involve a loss of such regulatory balance, reducing the ability to stabilize mood in the face of competing depression- and anxiety-related affective signals.

The common factor – reflecting general internalizing psychopathology – was consistently associated with both a lower affective baseline (i.e. affective setpoint) and greater gain-domain risk aversion. Regarding baseline mood, previous large-scale studies have established a reliable association between a lower affective setpoint and elevated depressive symptoms in both individuals scoring high on the BDI and patients diagnosed with MDD (Rutledge et al., 2017). Extending this work, our bifactor approach decomposed shared and specific symptom variance in depression and anxiety into three orthogonal components and revealed that only the common factor, rather than the depression- or anxiety-specific components, was significantly associated with a lower affective baseline. This finding suggests that the shared general distress component, rather than depression- or anxiety-specific symptom variance, is more closely related to individuals’ affective baselines (Kao et al., 2023). With respect to decision-making, prior literature using risky decision-making tasks without feedback has linked pathological anxiety to greater risk aversion (Charpentier et al., 2017). In line with this, our results from a risky decision-making task with feedback suggest that the common factor, rather than anxiety-specific variance per se, is more consistently associated with risk aversion. This suggests that heightened gain-domain risk aversion may be a transdiagnostic feature of internalizing psychopathology, rather than being uniquely attributable to anxiety. Notably, model comparison favored linear over nonlinear associations, indicating that even subclinical levels of general internalizing symptoms are measurably associated with affective baseline and gain-domain risk processing. Taken together, these findings underscore the importance of incorporating general internalizing dimensions into computational models of affect and choice.

Several limitations of the present study should be noted. First, although anxiety- and depression-related associations differed consistently, the anxiety-specific associations themselves were less robust across datasets. This pattern may raise the possibility of confounding by scale-related variability – that is, individual differences in how participants calibrate or use mood rating scales. Because both mood variability and $\beta _{RPE}$ are derived from subjective ratings, such scale-use differences could disproportionately affect weaker anxiety-specific effects. Our additional analyses, including analyses of two publicly available datasets with similar paradigms (Vanhasbroeck et al., 2021, n=49; Rutledge et al., n=46,204), did not support this explanation (see Appendix 3). Nevertheless, future studies would benefit from task designs that better control for scale sensitivity and elicit stronger mood responses to risky outcomes, possibly by incorporating more emotionally salient or high-stakes decision contexts. Second, our symptom assessment focused specifically on anxiety and depression. This choice was motivated by our primary hypotheses, but it limits our ability to evaluate the specificity of the observed associations. Recent work has shown that individuals with suicidal thoughts and behaviors exhibit reduced mood sensitivity to CR, but not to RPEs (Wang et al., 2026), suggesting that the current RPE-related effects are not driven by suicide-related processes. However, because we did not assess other psychiatric dimensions, such as compulsivity or schizophrenia-spectrum symptoms, we cannot determine whether the current findings are specific to anxiety- and depression-related symptom dimensions or instead reflect broader transdiagnostic psychopathology or nonspecific response-related variance. Future studies should include measures covering a wider range of psychiatric dimensions, such as internalizing, externalizing, inattentive/neurodevelopmental, mood/anxiety, and withdrawal dimensions identified by Wise et al., 2026, to better characterize whether links among symptom dimensions, RPE sensitivity, and mood variability are disorder-specific or transdiagnostic.

In conclusion, our findings emphasize the distinct associations of depression and anxiety with mood fluctuations, underlining the importance of partitioning shared and specific symptom variance in affective science. In particular, the robust association between depression and reduced RPE-related mood sensitivity may inform the development of computationally informed, mechanism-based interventions for affective disorders, offering a promising direction for future research and clinical translation.

## Materials and methods

A total of 2634 participants via online platforms (questionnaires from https://www.wjx.cn and tasks from https://www.naodao.com) took part in five experiments, including a psychometric experiment, a laboratory experiment, and two online replication experiments. Participants were recruited through participant pools and study advertisement. For online experiments, interested participants accessed the study through an online link and completed the questionnaires and task remotely. For the laboratory experiment, participants completed the study in a controlled laboratory setting. The study was approved by the Ethics Committee of Beijing Normal University (approve number: ICBIR_A_0016_028). Written or electronic informed consent was obtained from all participants before participation. Participants were paid a basic participation fee and a performance-based bonus, and the payment structure was explained before the experiment.

### Participants

Data from 1145 participants was collected in the psychometric experiment. Participants were excluded if (1) they failed any of the attentional checks (four items); (2) they made the same choices for all items; (3) they responded with extreme inconsistency in two similar questionnaires (difference in z-scores out of ± 2). The final sample for the psychometric dataset consisted of 901 participants. We recruited 59, 1087, and 343 participants for the laboratory experiment, the online experiment 1, and the online experiment 2, respectively. Participants were excluded if (1) they failed any of the attentional checks in questionnaires (four items), (2) they failed any of the catch trials (four trials), and (3) they responded too fast (reaction time for decision <200 ms) in more than 10% of the 90 trials. The final sample for the laboratory dataset, the online dataset 1, and the online dataset 2 included 44, 747, and 235 participants, separately. See Table 1 for demographic information and data collection periods. Because online data collection may raise concerns about AI-generated responses, we note that artificial intelligence tools, such as ChatGPT, became widely known to the public in November 2022, whereas all online experiments in the present study were conducted before November 2022 (see Table 1). Therefore, these data were unlikely to have been substantially affected by participants’ use of AI tools.

### Measurements of anxiety and depression

In line with previous literature orthogonally decomposing anxiety and depression (Gagne et al., 2022; Gagne et al., 2020), participants completed a set of Chinese version questionnaires of anxiety and depression. These measurements included the Mood and Anxiety Symptoms Questionnaire (MASQ; 62 items; Liu et al., 2015), the Trait subscale of the State-Trait Anxiety Inventory (TAI; 20 items; Shek, 1988), the BDI (21 items; Shek, 1990), the Penn State Worry Questionnaire (PSWQ; 16 items; Zhong et al., 2009), the Center for Epidemiologic Studies Depression Scale (CESD; 20 items; Jiang et al., 2019), and the Big Five Inventory-2 (BFI; 60 items; Zhang et al., 2022). Each item in the TAI, BDI, and CESD was rated on a four-point Likert scale, while a five-point Likert rating scale was used for the MASQ, PSWQ, and BFI. There were four items for attentional checks, which required the participants to make a specific choice and were embedded in the entire measurements, for example “please select the second option for this item”.

### Patients with affective disorders

We also recruited 121 patients with affective disorders, including MDD, anxiety disorder, and bipolar disorder. After excluding participants with no variance in mood ratings, the final sample included 116 patients. See Table 1, Appendix 1—table 8 for details.

### Experimental procedure

Participants were asked to make a choice between a certain option and a gamble (50% probability for each outcome) and to rate their momentary moods. Before the task protocol, participants were asked to rate their current happiness that we consider as their initial mood. At the beginning of the task, participants were endowed with 500 points. Each trial started with two options (a gamble option and a certain option) that presented randomly on each side (Figure 1F). Upon response, the chosen option would be highlighted in yellow for 0.5 s. Then the corresponding outcome at the screen center was presented for 1 s, followed by a fixation cross with a random duration (0.6~1.4 s). If the gamble was chosen, participants had equal probability to obtain each outcome. The obtained outcome would be accumulated to their total score, which was presenting at the top-right concern. Every two to three trials, participants rated “how happy are you at this moment” from 0 (very unhappy) to 100 (very happy) by moving a slider anchoring at midpoint (i.e. 50). Upon identifying their current mood, a fixation cross was presented with a random duration (0.6–1.4 s). Please note that the slider in the laboratory experiment was anchoring at the midpoint, that is 50. To exclude the potential anchoring effect, we did not set an anchor in the online replication experiment to check the robustness of our findings. This task consisted of 90 randomly presented trials, including 30 mixed trials, 30 gain trials, and 30 loss trials. In mixed trials, participants made a choice between a certain amount 0 and a gamble with a gain amount {40, 45, or 75} and a loss amount determined by a multiplier {0.2, 0.34, 0.5, 0.64, 0.77, 0.89, 1, 1.1, 1.35, or 2} on the gain amount. In gain trials, there was a certain gain amount {35, 45, or 55} and a gamble with 0 and a gain amount determined by a multiplier {1.68, 1.82, 2, 2.22, 2.48, 2.8, 3.16, 3.6, 4.2, or 5} on the certain gain amount. In loss trials, there were a certain loss amount {−35,–45, or –55} and a gamble with 0 and a loss amount determined by a multiplier {1.68, 1.82, 2, 2.22, 2.48, 2.8, 3.16, 3.6, 4.2, or 5} on the certain loss amount. Many amounts and multipliers were used to accommodate a wide range of risk and loss sensitivity, as in previous literature (Rutledge et al., 2014; Rutledge et al., 2015). We also set four trials embedded in the entire task for attentional checks. For example, participants were asked to make a choice between a certain gain 20 and a gamble 35/55, where the correct response for this trial was the gamble choice. All experimental procedures were programmed using Psychopy3 (2021.2.3) builder and hosted on https://www.naodao.com.

### Model fitting

We fit model parameters by using the method of maximum likelihood estimation (MLE) with fmincon function of MATLAB (version R2015a) at the individual level. To avoid local minimum, we ran this optimization function with random starting locations 50 times. Bayesian information criteria (BIC) were used to compare model fits.

### Mediation model

The mediation model was conducted using a sequence of regression steps. First, a regression of the independent variable depression on the dependent variable mood variation was performed; Second, a regression of the independent variable depression on the mediator variable $\beta _{RPE}$ was performed; Next, a regression of the independent variable depression and the mediator variable $\beta _{RPE}$ on the dependent variable mood variation was performed; Finally, the indirect and total effects were estimated.

### Statistical analysis

We performed correlations among depression/anxiety factor scores, behavioral indices, and parameters using Matlab R2015a. To further validate our main correlational results, the percentile bootstrap CIs were estimated using the R package ‘boot’ and 5000 bootstrap resamples. We used an online calculator (https://www.psychometrica.de/correlation.html) to examine differences between two correlation coefficients. All reported tests are two-tailed. We set the significance level at *P*=0.05.

## Data Availability

The data and code that support the findings of this study are available from https://github.com/ZhihaoWangpsyer/depression_anxiety_mood (copy archived at Wang, 2026).

## Additional files

MDAR checklist

## Appendix 1

### Psychometric properties of the bifactor model of anxiety and depression in the Chinese population

We tested the validation of the tripartite model of anxiety and depression in the Chinese population (n=901). All participants were asked to complete a set of anxiety- and depression-related questionnaires (see Materials and methods), as previous literature on the tripartite model (Bentham, 1780; Mill, 1859). To examine the underlying factor structure, the entire sample was equally divided into two datasets according to the participants’ number for exploratory and confirmatory factor analysis (EFA dataset, the first part, 1–450; CFA dataset, the second part, 451–901), respectively. For the EFA dataset, we determined the number of factors based on theoretical considerations (i.e. the tripartite model of anxiety and depression Eldar and Niv, 2015; Kao et al., 2023; Emanuel and Eldar, 2023) and scree plot. Results showed that our data was best explained by three factors (40.24%). We then used the three-factor bifactor model to decompose the item-level covariance matrix into a general factor and two specific factors. The ‘Psych’ package in R was used to perform Schmid-Leiman (SL) orthogonalization procedure to obtain factor loadings for each item. Specifically, the SL procedure used oblique factor analysis and higher order factor analysis based on the lower order factor correlations to extract the shared higher order factor (the common factor). Factor scores for each participant were calculated using the Anderson-Rubin method, which is a weighted-least squares solution that maintains the orthogonality of the common score and each specific factor score. Results of factor loadings indicated that the common factor had high loadings (>0.4) for multiple anxiety-related and depression-related items and moderately high loadings (>0.2) across almost all items (Figure 1D). One specific factor had high loadings (>0.4) on items related to anhedonia while another specific factor had high loadings (>0.4) on items related to worry and anxious arousal (Figure 1D).

In the CFA dataset, all items were allowed to load onto the common factor. Each item was also allowed to load onto either the anxiety-specific factor or the depression-specific factor. The assignment for each item was depended on whether the loading on each specific factor was greater than 0.2 in the EFA dataset. After item assignments, factor loadings were re-estimated with diagonally weighted least squares estimation (DWLS). This method only allowed each item to load on the loaded factor in the EFA dataset (>0.2), because it is more appropriate for ordinal data and less sensitive to deviations from normality, as compared with maximum likelihood estimation (Bennett et al., 2022). We implemented this procedure with the specification of an orthogonal solution using the ‘Lavaan’ package in R. The quality of the fit was evaluated by the comparative fit index (CFI), Tucker-Lewis index (TLI), root mean square error of approximation (RMSEA), and standardized root-mean-square residual (SRMR). Results showed excellent goodness-of-fit indices (CFI = 0.989, TLI = 0.989, RMSEA = 0.046, SRMR = 0.061). We additionally performed the same analysis for the CFA dataset as in the EFA dataset to access the similarity between different datasets. That is, we conducted an unconstrained (i.e., exploratory, but not confirmatory) bifactor analysis in the CFA dataset. The resulting factor loadings were highly congruent with the factor loadings in the EFA dataset (cosine-similarity was 0.99 for the common factor loadings, 0.99 for the depression-specific factor loadings, and 0.98 for the anxiety-specific factor loadings). As predicted by the factor structure, the common factor scores are orthogonal to each specific factor score (Figure 1C&E).

To verify the factor structure underlying anxiety and depression, our model space also included a three-factor high-order model and a simple three-factor model. Comparison of model fit revealed that the bifactor model outperformed other models (Appendix 1—table 1), confirming the bifactor structure underlying anxiety and depression. In addition, the common factor showed high correlations with all questionnaires (*r*s >0.69; Figure 1F), except the anxious arousal subscale of the Mood and Anxiety Symptoms Questionnaire (MASQaa; *r*=0.16). The anxiety-specific factor was mainly contributed by the anxiety subscale of the State-Trait Anxiety Inventory (TAIanx, *r*=0.60), the neuroticism of the Big Five Inventory-2 (BFIn, *r*=0.41), the Penn State Worry Questionnaire (PSWQ, *r*=0.41), MASQaa (*r*=0.86), the Beck Depression Inventory (BDI; *r*=0.52), and the Center for Epidemiologic Studies Depression Scale (CESD; *r*=0.54), while the depression-specific factor was mainly contributed by the depression subscale of the State-Trait Anxiety Inventory (TAIdep, *r*=0.52) and the anhedonia depression subscale of the Mood and Anxiety Symptoms Questionnaire (MASQad; *r*=0.58). Correlations between factors and questionnaires showed the same pattern in different datasets (Figure 1—figure supplement 6). These results indicated good construct validity of this bifactor model of anxiety and depression. Together, these results validated the bifactor model of anxiety and depression in the Chinese population and supported the classic tripartite model. Regarding the application, the bifactor model was initially derived and validated using the larger psychometric dataset (n=901). For subsequent analyses, we applied this validated bifactor solution by computing factor scores using the loadings obtained from the original psychometric sample. This operation was consistent with recent literature of computational psychiatry (Eldar et al., 2016).

In addition, the ratio of 450 participants to 128 items in the EFA split-half sample, approximately 3.5:1, is below some conventional sample-size recommendations for EFA, including the often-cited recommendation of five participants per item (Rutledge et al., 2014). However, the split-half EFA yielded a stable factor structure, and the independent CFA further supported the robustness of this solution. Moreover, reliable EFA solutions may be obtained even with relatively small samples when the data are well-conditioned, such as when factor loadings are high, the number of factors is small, and each factor is defined by multiple items (Otto et al., 2016). These conditions were largely met in our data.

For factor score extraction, we followed prior work using bifactor modeling in computational psychiatry (Rutledge et al., 2015) and extracted factor scores with the Anderson–Rubin method, implemented using psych::factor.scores with method = "Anderson". This approach yields standardized and mutually orthogonal factor scores, which is particularly appropriate for our subsequent correlation analyses because it avoids multicollinearity among the general, depression-specific, and anxiety-specific factors. As a robustness check, we also extracted factor scores using the Bartlett method from an oblique bifactor model, which allowed the depression- and anxiety-specific factors to correlate. In the combined dataset (n=1026), anxiety- and depression-specific scores were significantly correlated when extracted using the Bartlett method (*r*=0.638, p<0.001), whereas, as expected, they were effectively uncorrelated when extracted using the Anderson–Rubin method (*r*<0.001, p=1.000). This comparison suggests that the Anderson–Rubin method is more appropriate for our analytic aim of isolating the unique contributions of shared and symptom-specific variance because it separates the general distress/internalizing factor from residual anxiety- and depression-specific components. Thus, we retained the Anderson–Rubin factor scores in the main analyses. We further analyzed non-orthogonalized full depression and anxiety scores to assess the robustness of our results. When full depression and anxiety scores were entered in separate linear mixed-effects models predicting RPE-related mood sensitivity, with dataset included as a random intercept, the anxiety association was not significant (anxiety: b=0.002, *t*=1.130, p=0.259; depression: b=–0.008, *t*=–3.732, p<0.001). By contrast, when the full non-orthogonalized anxiety and depression scores were entered simultaneously in the same linear mixed-effects model, the original pattern was replicated: anxiety and depression showed opposing associations with RPE-related mood sensitivity (anxiety: b=0.012, *t*=4.619, p<0.001; depression: b=–0.017, *t*=–5.851, p<0.001). This pattern is consistent with a mutual suppression effect: shared variance between anxiety and depression may obscure their unique associations when examined separately, whereas simultaneous regression reveals their opposing symptom-specific associations. These results support our interpretation that RPE-related mood sensitivity is linked to the separable anxiety- and depression-specific components.

**Appendix 1—table 1.** Psychometric model comparisons.

Models	CFI	TLI	RMSEA	SRMR
Bifactor model	0.989	0.989	0.046	0.061
Three-factor model	0.794	0.789	0.200	0.205
High-order model	0.981	0.981	0.060	0.072

**Appendix 1—table 2.** Mood model comparisons.

Model number	# of parameters	Δ BIC			
		Laboratory dataset N=44	Online dataset 1 N=747	Online dataset 2 N=235	Clinical dataset N=116
Model #1	6	0	0	0	0
Model #2	5	43.99	918.62	228.00	41.57
Model #3	8	122.13	1815.15	586.31	549.73
Model #4	7	155.22	2662.46	814.28	424.10
Model #5	7	82.24	709.23	204.32	151.56
Model #6	6	47.76	2514.71	743.17	601.16

Δ BIC, Bayesian information criterion relative to the winning model (Model #1).

**Appendix 1—table 3.** Objective vs. subjective happiness models.

	# of parameters	Δ BIC			
		Healthy dataset N=1026	Clinical dataset N=116	Vanhasbroeck et al., dataset N=49	Rutledge’s dataset N=46,204
Model #1	6	0	0	0	0
Model #6	5	3305.64	601.16	118.00	4997.29
Model #1 vs 6 EP	-	1.00	1.00	1.00	1.00

Δ BIC, Bayesian information criterion relative to the winning model (Model #1); EP, exceedance probability; EP greater than 0.95 was considered significant.

**Appendix 1—table 4.** Time effect on happiness.

Model number	# of parameters	Δ BIC			
		Healthy dataset N=1026	Clinical dataset N=116	Vanhasbroeck et al., dataset N=49	Rutledge’s dataset N=46,204
Model #1	6	0	0	0	0
Model #7	6	1883.39	84.88	98.15	4152.61
Model #8	6	1487.79	66.37	61.73	2295.40
Model #9	6	1064.22	59.53	74.40	1458.34

Δ BIC, Bayesian information criterion relative to the winning model (Model #1).

**Appendix 1—table 5.** Contributions to model fit.

Model number	# of parameters	Mean R^2^			
		Healthy dataset N=1026	Clinical dataset N=116	Vanhasbroeck et al., dataset N=49	Rutledge’s dataset N=46,204
M1: CR +EV + RPE	6	0.68	0.47	0.58	0.69
M10: No CR	5	0.62	0.42	0.52	0.60
M11: No EV	5	0.59	0.40	0.54	0.60
M12: No RPE	5	0.42	0.29	0.34	0.50

**Appendix 1—table 6.** Interaction between different wins and risk preference for gain on z-scored happiness.

Datasets	Trial types	
	Certain trials	Gamble trials (better outcomes; nonzero)
Healthy dataset N=1026	beta = –0.00; t=–0.07; p=0.943 95% CI = [–0.01, 0.01]	beta = –0.00; t=–0.24; p=0.807 95% CI = [–0.00, 0.00]
Clinical dataset N=116	beta = –0.01; t=–0.64; p=0.524 95% CI = [–0.04, 0.02]	beta = –0.00; t=–2.13; p=0.034 95% CI = [-0.01,–0.00]
Vanhasbroeck et al., dataset N=49	beta = 1.41; t=0.51; p=0.609 95% CI = [–4.00, 6.82]	beta = 0.38; t=0.55; p=0.581 95% CI = [–0.96, 1.71]
Rutledge’s dataset N=46,204	beta = 0.10; t=0.84; p=0.402 95% CI = [–0.14, 0.34]	beta = 0.03; t=0.82; p=0.412 95% CI = [–0.04, 0.10]

**Appendix 1—table 7.** Correlations of the depression-specific factor with mood variation and βRPE.

	Laboratory dataset	Online dataset 1	Online dataset 2	Clinical dataset
Mood variation	*r*=–0.309 p=0.041	*r*=–0.191 p<0.001	*r*=–0.135 p=0.039	*r*=–0.239 p=0.009
β_RPE_	*r*=–0.352 p=0.019	*r*=–0.142 p<0.001	*r*=–0.189 p=0.004	*r*=–0.216 p=0.020
Mediation	a×b = –0.306 95% CI: [-0.547,–0.065] p=0.015	a×b = –0.096 95% CI: [-0.145,–0.047] p<0.001	a×b = –0.126 95% CI: [-0.212,–0.040] p=0.004	a×b = –0.141 95% CI: [-0.261,–0.038] p=0.021

**Appendix 1—table 8.** Clinical characteristics of patients with affective disorders.

Clinical variables	N=116
Diagnosis (MDD/AD/MDD and AD/others)	51/22/32/11
Illness duration (months)	19.57±19.22
Medications (yes)	
SSRI	96
Antipsychotics	57
BZDs	38
Mood stabilizer	14

Note that mood stabilizer refers to Lithium in this dataset.

**Appendix 1—table 9.** Choice model comparisons.

Model number	# of parameters	Δ BIC			
		Laboratory dataset N=44	Online dataset 1 N=747	Online dataset 2 N=235	Clinical dataset N=116
cM1	1	320.92	10959.49	3818.36	2368.27
cM2	4	62.70	3655.22	1030.07	620.85
cM3	6	0	0	0	0

Δ BIC, Bayesian information criterion relative to the winning model (cM3).

## Appendix 2

### Computational model of momentary mood

To quantify how different events impacted participants’ momentary moods during the gambling task, we used the classic model assuming that momentary mood depends on the recency-weighted average of the chosen certain reward (CR), expected value of the chosen gamble (EV), and reward prediction error (RPE; M1; Equation 2). RPE was defined as the difference between the obtained and expected value. We also incorporated a drift parameter to account for the gradual change in happiness over time (Rutledge et al., 2016a; Blain and Rutledge, 2020).

(2) $$\displaystyle  {\rm Happiness}\left (t\right)=\beta _{0}\mathrm{+}\beta _{CR}\sum _{j=1}^{t}\gamma ^{t-j}CR_{j}+\beta _{EV}\sum _{j=1}^{t}\gamma ^{t-j}EV_{j}+\beta _{RPE}\sum _{j=1}^{t}\gamma ^{t-j}RPE_{j}+\beta _{t}t$$

here, *t* and *j* are trial numbers, *β*_0_ is a baseline mood parameter, other weights *β* capture the influence of different event types. γ ∈ [0, 1] is a decay parameter explicitly modeling the influence of previous trials on current happiness ratings, assigning greater weight to recent events and progressively diminishing influence to earlier trials. CR*_j_* is the CR if the certain option was chosen on trial *j*; otherwise, CR*_j_* is 0. EV*j* is the EV and RPEj is the RPE on trial *j* if the gamble was chosen. If the certain option was chosen, then EV*_j_* = 0 and RPE*j* = 0.

Although M1 has been shown to accurately track mood data, we also fit other candidate mood models. Specifically, to verify the notion that momentary moods depend on RPE in addition to reward expectation, we fit an alternative model in which moods are contributed by the recency-weighted average of CR and the gamble reward (GR; M2; Equation 3).

(3) $$\displaystyle  {\rm Happiness}\left (t\right)=\beta _{0}+\beta _{CR}\sum _{j=1}^{t}\gamma ^{t-j}CR_{j}+\beta _{GR}\sum _{j=1}^{t}\gamma ^{t-j}GR_{j}+\beta _{t}t$$

To verify that mood ratings are best explained by a shared forgetting factor (i.e. the recency-weighted history of different event types), we compared a model with a single decay parameter to an alternative model including forgetting factors for each event type, e.g., different decay parameters for CR, EV, and RPE (M3; Equation 4).

(4) $$\displaystyle  {\rm Happiness}\left (t\right)=\beta _{0}+\beta _{CR}\sum _{j=1}^{t}\gamma _{CR}^{t-j}CR_{j}+\beta _{EV}\sum _{j=1}^{t}\gamma _{EV}^{t-j}EV_{j}+\beta _{RPE}\sum _{j=1}^{t}\gamma _{RPE}^{t-j}RPE_{j}+\beta _{t}t$$

It has been shown that moods are influenced by automatically comparing alternative outcomes. We thus considered a component of the outcome difference (OD) between the chosen option and unchosen option (M4; Equation 5) and a regret (R) component (upward comparison; M5; Equation 6) based on M1.

(5) $$\displaystyle  {\rm Happiness}\,(t) = \beta_0 + \beta_{CR}\sum_{j=1}^{t} \gamma^{t-j}CR_j + \beta_{EV}\sum_{j=1}^{t} \gamma^{t-j}EV_j + \beta_{RPE}\sum_{j=1}^{t} \gamma^{t-j}RPE_j + \beta_{OD}\sum_{j=1}^{t} \gamma^{t-j}OD_j + \beta_t t$$

where OD is the outcome difference between the chosen option and the unchosen option.

(6) $$\displaystyle  {\rm Happiness}\left (t\right)=\beta _{0}+\beta _{CR}\sum _{j=1}^{t}\gamma ^{t-j}CR_{j}+\beta _{EV}\sum _{j=1}^{t}\gamma ^{t-j}EV_{j}+\beta _{RPE}\sum _{j=1}^{t}\gamma ^{t-j}RPE_{j}+\beta _{R}\sum _{j=1}^{t}\gamma ^{t-j}R_{j}+\beta _{t}t$$

where R represents how much better the outcome could have been if the other option had been chosen. Mathematically, R is the difference between the obtained outcome and the potential better outcome if it exists; otherwise, *R*=0. We also considered subjective values, rather than objective values in M1, to model mood, as to calibrate the rating scale that participants may use (see Appendix 7 for details).

In the healthy datasets, we conducted a linear mixed-effect model against decay parameter (gamma) with all three factors, with dataset as a random factor. Results did not show significant effect (common: *t*=0.708, p=0.479; anxiety: *t*=0.564, p=0.573; depression: *t*=1.146, p=0.252). In the clinical dataset, we conducted a linear model against decay parameter (gamma) with all three factors and found no significant effect (common: *t*=–0.036, p=0.972; anxiety: *t*=–0.047, p=0.963; depression: *t*=0.052, p=0.959).

## Appendix 3

### Calibration of rating scales

There was an important issue that individual differences in risk preference may affect the subjective calibration of mood rating scales. Specifically, individuals with nonlinear utility functions (e.g. risk-seeking participants) could display disproportionately high mood responses for large relative to small wins, potentially affecting model interpretation. To systematically address this issue, we conducted several complementary analyses:

*First*, we examined whether objective or subjective utility models better explained happiness ratings. To enhance statistical power and simplify interpretation, we combined our laboratory and two online datasets into a single healthy dataset. Model comparisons consistently favored the objective happiness model (M1) over the subjective model (M6), as indicated by lower Bayesian Information Criterion (BIC) values and significantly higher exceedance probabilities (EP >0.95, Appendix 1—table 3), suggesting the objective model better captured happiness ratings at both individual and group levels.

*Second*, within the winning model (objective one), we replicated opposed roles of RPE in mood dynamics of depression and anxiety after controlling for risk attitudes (ps <0.009).

*Third*, we explicitly assessed whether risk preferences moderated the relationship between wins and mood ratings. Linear mixed-effects analyses on z-scored happiness with subject as a random factor and with different wins and risk preference for gain showed no significant interaction effects between risk preference and win size on happiness ratings for either certain or gamble trials (all *p*-values >0.05, Appendix 1—table 6).

*Fourth*, we validated these findings externally by examining two open datasets using similar risk-based decision-making tasks with repeated happiness ratings: (Vanhasbroeck et al., 2021; n=49) and Rutledge’s smartphone App dataset (n=46,204). Consistent with our own data, model comparisons again favored the objective happiness model (Appendix 1—table 3), and interaction analyses revealed no meaningful moderation by risk preferences (Appendix 1—table 6).

*Finally*, we fitted a simple happiness model with unit wins and losses (Equation 7; mean R2=0.65 for healthy dataset and 0.43 for clinical dataset). Mood sensitivity parameters were not significantly correlated with individual differences in risk preferences, either in our healthy (wins: *r*=0.017, p=0.588; losses: *r*=0.009, p=0.765) or clinical datasets (wins: *r*=0.072, p=0.441; losses: *r*=0.014, p=0.882).

(7) $$\displaystyle  {\rm Happiness}\left (t\right)=\beta _{0}+\beta _{win}\sum _{j=1}^{t}\gamma ^{t-j}win_{j}+\beta _{loss}\sum _{j=1}^{t}\gamma ^{t-j}loss_{j}+\beta _{t}t$$

Collectively, these comprehensive analyses suggest that individual differences in risk preference did not significantly affect the calibration of mood ratings, or alter our primary findings regarding distinct mood dynamics in anxiety versus depression. Instead, these results possibly suggest dissociable processes for decision-making (i.e. value perception) and mood dynamics.

## Appendix 4

### Orthogonality of happiness weights on CR, EV, and RPE

One potential concern was the orthogonality of happiness weights on CR, EV, and RPE. Indeed, we found significant correlation between them (healthy dataset: 0.348<rs < 0.625, ps <0.001; clinical dataset: 0.260<rs < 0.679, ps <0.005). Parameter recovery analysis showed high correlations between the matched simulated and real parameters (healthy dataset: 0.818<rs < 0.943, ps <0.001; clinical dataset: 0.815<rs < 0. 955, ps <0.001) and low correlations between the mismatched simulated and real parameters (healthy dataset: 0.269<rs < 0.572, ps <0.001; clinical dataset: 0.172<rs < 0. 635, ps <0.065; Figure 1—figure supplement 4), confirming the identification of these parameters. To examine whether risk preferences influence their orthogonality, correlation analyses did not show significant correlation between risk preferences and model fit (e.g. R^2^; healthy dataset: abs(r)<0.042, ps >0.182; clinical dataset: abs(r)<0.105, ps >0.261). When controlling for risk attitudes and model fit, our findings of opposed roles of RPE in mood dynamics of depression and anxiety can be replicable (healthy dataset: p*s* <0.002; clinical dataset: for depression: p=0.016). In addition, we fitted a simple happiness model with unit wins and losses (Equation 7; mean R2=0.65 for healthy dataset and 0.43 for clinical dataset). Mood sensitivity parameters were not significantly correlated with individual differences in risk preferences, either in our healthy (wins: *r*=0.017, p=0.588; losses: *r*=0.009, *P*=0.765) or clinical datasets (wins: *r*=0.072, p=0.441; losses: *r*=0.014, p=0.882). These results suggest that the current parameters can be identified and individual differences in risk attitudes do not influence parameter recovery.

## Appendix 5

### Time effect on happiness

Although we incorporated a drift parameter to account for the gradual change in happiness over time (β_t_: t=–5.84, p<0.001), time may also interact with event parameters, affecting mood sensitivity dynamically. Therefore, we tested three additional models explicitly incorporating interactions between time-on-task and different event parameters (Models M7–M9; see Equations 8–10), enabling us to systematically evaluate how mood responses to events might change with time.

(8) $$\displaystyle  {\rm Happiness}\left (t\right)=\beta _{0}+\beta _{CR}\sum _{j=1}^{t}\gamma ^{t-j}CR_{j}+\beta _{EV}\sum _{j=1}^{t}\gamma ^{t-j}EV_{j}+\beta _{RPE}\sum _{j=1}^{t}\gamma ^{t-j}RPE_{j}+\beta _{t}\sum _{j=1}^{t}\gamma ^{t-j}CR_{j}t$$

(9) $$\displaystyle  {\rm Happiness}\left (t\right)=\beta _{0}+\beta _{CR}\sum _{j=1}^{t}\gamma ^{t-j}CR_{j}+\beta _{EV}\sum _{j=1}^{t}\gamma ^{t-j}EV_{j}+\beta _{RPE}\sum _{j=1}^{t}\gamma ^{t-j}RPE_{j}+\beta _{t}\sum _{j=1}^{t}\gamma ^{t-j}EV_{j}t$$

(10) $$\displaystyle  {\rm Happiness}\left (t\right)=\beta _{0}+\beta _{CR}\sum _{j=1}^{t}\gamma ^{t-j}CR_{j}+\beta _{EV}\sum _{j=1}^{t}\gamma ^{t-j}EV_{j}+\beta _{RPE}\sum _{j=1}^{t}\gamma ^{t-j}RPE_{j}+\beta _{t}\sum _{j=1}^{t}\gamma ^{t-j}RPE_{j}t$$

Model comparisons consistently favored the simpler intercept-only drift model (M1) over these interaction models (M7–M9), as indicated by lower Bayesian Information Criterion (BIC) values (Appendix 1—table 4). Furthermore, we validated these results externally using two publicly available datasets with similar paradigms (Vanhasbroeck et al., 2021; n=49; and Rutledge’s smartphone app dataset, n=46,204). These external validations also strongly favored the simpler drift model without time-event interactions (see Appendix 1—table 4). Taken together, these analyses suggest that while overall mood indeed exhibits gradual drift over time, there is minimal evidence to support time-on-task by event interactions substantially affecting mood sensitivity to event parameters in our task, supporting the robustness and generalizability of our primary findings.

## Appendix 6

### Contributions to model fit

To elaborate on which components (CR, EV, or RPE) contributed most to model fit, we tested three additional models. Specifically, based on the winning model (M1), we kicked out each event component separately (M10-12, see Equations 11–13).

(11) $$\displaystyle  {\rm Happiness}\left (t\right)=\beta _{0}+\beta _{EV}\sum _{j=1}^{t}\gamma ^{t-j}EV_{j}+\beta _{RPE}\sum _{j=1}^{t}\gamma ^{t-j}RPE_{j}+\beta _{t}t$$

(12) $$\displaystyle  {\rm Happiness}\left (t\right)=\beta _{0}+\beta _{CR}\sum _{j=1}^{t}\gamma ^{t-j}CR_{j}+\beta _{RPE}\sum _{j=1}^{t}\gamma ^{t-j}RPE_{j}+\beta _{t}t$$

(13) $$\displaystyle  {\rm Happiness}\left (t\right)=\beta _{0}+\beta _{CR}\sum _{j=1}^{t}\gamma ^{t-j}CR_{j}+\beta _{EV}\sum _{j=1}^{t}\gamma ^{t-j}EV_{j}+\beta _{t}t$$

The results showed that removing RPE led to the most substantial drop in model fit: ΔR²=0.26 in the healthy dataset and ΔR²=0.18 in the clinical dataset (Appendix 1—table 5). This indicates that RPE was the most important contributor to explaining mood dynamics. In contrast, excluding either CR or EV resulted in only modest reductions in model fit (ΔR²<0.09 in the healthy dataset and ΔR²<0.07 in the clinical dataset). We replicated this pattern in two independent, publicly available datasets (Vanhasbroeck et al., 2021, n=49; and Rutledge’s smartphone app dataset, n=46,204), further supporting the robustness and generalizability of the central role of RPE in momentary mood computations (Appendix 1—table 5). These analyses confirm that reward prediction error is the dominant predictor of trial-by-trial mood changes.

## Appendix 7

### Computational model of gambling choice

To quantify how different events impacted participants’ momentary moods during the gambling

In line with previous studies (Quoidbach et al., 2019; Taquet et al., 2016), our choice model space included expected value model (cM1), prospect theory model (cM2) (Broome et al., 2015), and approach-avoidance prospect theory model (cM3) (Quoidbach et al., 2019). For cM2 (Equations 14–17), there were 3 parameters, including risk aversion (α, range: [0.3, 1.3]), loss aversion (*λ*: [0.5, 5]), and inverse temperature (*μ*: [0, 10]).

(14) $$\displaystyle U_{gamble}=0.5\left (V_{gain}\right)^{\alpha}-0.5\lambda \left (-V_{loss}\right)^{\alpha}$$

(15) $$\displaystyle U_{certain}=\left (V_{certain}\right)^{\alpha}\,if\,V_{certain}\geq 0$$

(16) $$\displaystyle U_{certain}=-\lambda \left (-V_{certain}\right)^{\alpha}\,if\,V_{certain}{\lt}0$$

(17) $$\displaystyle P_{gamble}=\frac{1}{1+e^{-\mu \left (U_{gamble}-U_{certain}\right)}}$$

where V*_gain_* and V*_loss_* are the objective gain and loss from a gamble, respectively. Please note that V*_gain_* is 0 in loss trials and V*_loss_* is 0 in gain trials. V*_certain_* is the objective value for the certain option. U*_gamble_* and U*_certain_* denote the subjective utilities of the gamble and the certain option, respectively. Choice probability for gamble (P*_gamble_*) is determined by the softmax rule. Building on cM2, cM3 decomposes the decision process into risk-attitude-driven valuation (e.g. loss and risk aversion) and value-insensitive motivational components (Equations 14–16; 18–20). That is, choice probability for P*_gamble_* in cM3 is jointly determined by the softmax rule and approach/avoidance parameters ($\beta _{gain}$: [–1, 1], $\beta _{loss}$: [–1, 1]). Approach/avoidance parameters are not applied in mixed trials. Please note that a higher gambling rate does not imply a change in risk attitude per se: it can arise from an increased value-insensitive approach bias even when risk-attitude parameters are comparable between groups. Risk attitude is indeed conceptualized in economics as the curvature of the utility function (i.e. the subjective value) of the objective outcomes, with concave curves associated with risk aversion, and convex curves associated with risk seeking (Mason et al., 2017; Taquet et al., 2020). By contrast, the approach or avoidance bias applies to all the value. A possible interpretation of the approach bias is that participants approach the option with the highest possible gain (the lottery) in the gain frame; the avoidance bias would then reflect a tendency to systematically avoid the highest potential losses (the lottery) in the loss frame.

(18) $$\displaystyle P_{gamble}=\frac{1-\beta _{val}}{1+e^{-\mu \left (U_{gamble}-U_{certain}\right)}}+\beta _{val}\quad if\,\beta _{val}\geq 0$$

(19) $$\displaystyle P_{gamble}=\frac{1+\beta _{val}}{1+e^{-\mu \left (U_{gamble}-U_{certain}\right)}}\quad if\,\beta _{val}{< }0$$

(20) $$\displaystyle \beta_{val} \begin{cases} \beta_{gain}, & {gain\, trials},\\ \beta_{loss}, & {loss\, trials}. \end{cases}$$

Model comparison using BIC revealed that the winning model for each dataset was the approach-avoidance prospect theory model (cM3; mean R^2^=0.51 for the laboratory dataset, 0.49 for the online dataset1, 0.54 for the online dataset 2, and 0.40 for the clinical dataset; Appendix 1—table 9).
