# Supplementary material for: Dissociable roles of reward prediction error in the contrasting mood dynamics of depression and anxiety
Source: eLife. 2026 Sep 29;15:RP110631. doi: 10.7554/eLife.110631 (PMC13623375; doi:10.7554/eLife.110631)
Supplement: Peer review history

# Editor report: eLife Assessment

**DOI:** 10.7554/eLife.110631.3.sa0

**Authors:** Andreea Oliviana Diaconescu

**Affiliations:** University of Toronto Canada

**Keywords:** Compelling, Important

This is an **important** study that uses a tripartite transdiagnostic framework to separate depression-specific, anxiety-specific, and shared psychopathology dimensions and relate them to mood variability and mood reactivity to reward prediction errors across several large non-clinical cohorts and a clinical sample. The evidence is **compelling**: large samples, a well-characterised gambling task, rigorous computational and psychometric analyses (i.e., split-half replication of the factor structure, convergent results with non-orthogonalised factors, an explicitly specified risk-attitude model, diagnostic breakdown and power analyses for the clinical cohort) and replication of the depression-specific blunting of reward prediction error sensitivity in patients. Anxiety-specific associations emerge reliably only when data are pooled and are likely underpowered clinically given co-morbid anxious depression, a constraint the authors now state explicitly. The work advances a mechanistic account of how distinct symptom dimensions shape reward-based mood updating.

# Referee report: Reviewer #1 (Public review)

**DOI:** 10.7554/eLife.110631.3.sa1

Summary of strengths:

Thank you very much for giving me the opportunity to review this very interesting paper. The research question is intriguing, allowing to address commonly observed co-morbidities between depression and anxiety and their dissociable and opposite relationship to mood fluctuations and sensitivity to reward prediction errors. The computational analyses are very in-depth, including many state of the art checks and validations. Finally, another strength is the inclusion of several large or very large samples, including a patient sample in addition to the general population sample.

Comments on revised version.

I want to thank the authors for taking the time to answer all my questions. Their answers were very thoughtful and well argued. I found the theoretical explanations very helpful for explaining their approach and ideas further. In particular, it was fascinating to see how including a single non-orthogonalized depression or anxiety scored show no effect, but including them in simultaneously revealed their previously observed patterns.

# Referee report: Reviewer #2 (Public review)

**DOI:** 10.7554/eLife.110631.3.sa2

Summary:

Despite their common co-occurrence, depression and anxiety are known to alter mood fluctuations in opposite ways. Here, the authors aimed at distinguishing depression-specific from anxiety-specific from psychopathology-general effects of reward processing on mood fluctuations, focusing on reward prediction errors (RPE) which are known to be linked to mood fluctuations. This mechanistic study aims at uncovering the process through which these psychopathologies are associated with mood modulations. The authors were able to appropriately test their hypothesis and obtained results corroborating their conclusions.

This work provides a convincing demonstration of the relevance of computational psychiatry (Huys et al, 2016) and the use of decision neuroscience to shed light on the interplay of anxiety and depression and mood.

Comments on revised version.

(1) Methodological & Theoretical Framework: The authors used a tripartite model to effectively distinguish depression vs anxiety dimensions from broad psychopathology/distress.

(2) Possible theoretical confounds: This manuscript addressed adequately the concerns one would have regarding risk-attitudes.

(3) Computational Rigor: The computational model elegantly separates reward expectations (EV in the model) from outcome processing through RPE, which are two sequential cognitive processes, providing a fine-grained mechanistic account of mood fluctuations.

(4) Clear & Logical Results Structure: In response to feedback provided during the previous round of review (previously cited as a recommendation for authors), the authors re-organized the Results section into three distinct, easy-to-navigate subsections (Depression, Anxiety, and Depression vs. Anxiety), which substantially improves readability and clarity.

(5) Neurobiological Context: The Discussion has been enriched with a well-integrated overview of the neural circuits (striatal-midbrain dopaminergic, vmPFC, OFC, and anterior insula) likely underpinning RPE-driven mood updates, which is sure to improve the translational interest of this work.

(6) Transparent Reporting: The authors had already provided a trustworthy writing approach when referring to trending statistical results. In this revised manuscript, they have been exceptionally transparent regarding study limitations, data collection timelines (addressing potential AI-related artifacts), and statistical power constraints.

Status of Previous Weaknesses & Suggested Revisions

(1) Clinical Sample Size and Anxiety-Specific Effects

Previous Concern: The sample size of the clinical sample (N=116) may not be sufficient to detect anxiety-specific effects due to the high rate of comorbid anxious depression. It would be beneficial to include the number of MDD vs GAD vs anxious depression diagnoses in the clinical population as this would be likely to shine light on the power limitations.

- Author Revision: The authors have fully addressed this point by adding a diagnostic breakdown in Table S8 which details the diagnosis, illness duration, and medication status. They also included details of the power analysis (Discussion, pages 17-18) putting into perspective their results and provided two possible literature-informed interpretations of their findings. This is also reflected in their updated Abstract.

(2) Re-organization of Results

Previous Suggestion: The results sections 2 (depression) and 3 (anxiety) could be improved by reducing the back and forth between factors throughout the results. It may be useful to split them into 3 sections: depression only, anxiety only, depression vs anxiety.

- Author Revision: The Results section was restructured as suggested, cleanly isolating depression-specific associations, anxiety-specific associations, and direct statistical comparisons between the two ("differential associations" in the manuscript).

(3) Neurocircuitry Discussion

Previous Suggestion: In the discussion, authors could have mentioned the brain areas most likely to be involved in these processes, both cognitive and psychopathological, as previous studies (such as Cecchi et al, 2022) have aimed at identifying regions involved in RPE processing while modulating mood in health. A short section on this would be useful to the neuropsychiatric community.

- Author Revision: A concise section was added to the Discussion mapping computational parameters onto striatal, prefrontal, and insular circuitry (see Strengths #5).

Conclusion:

The authors have satisfactorily resolved all minor-to-moderate issues raised in the previous review.

# Referee report: Reviewer #3 (Public review)

**DOI:** 10.7554/eLife.110631.3.sa3

Summary:

In this submission Wang and colleagues jointly examine the association between depression and anxiety symptoms and individuals' affective reactivity to reward prediction errors in Ruttledge et al.'s gambling paradigm. Taking a bifactor approach to anxiety and depression in several non-clinical (and one clinical sample), the authors find that anxiety-specific symptoms relate to over-reactivity of mood to reward prediction errors (RPEs) as well as heightened mood variability , while depression-specific symptoms relate to blunted mood sensitivity to RPEs. These depression-, but not-anxiety specific relationships replicated in patient samples.

Strengths:

I was impressed that the data-driven, transdiagnostic approach employed by the authors uncovered specific relationships between anxiety and depression-specific factors and RPE reactivity in a well characterized task and computational model, especially in a non-clinical sample. This sheds new light on how these affective processes may be perturbed-and importantly, in different ways-by anxiety and depression symptoms. Likewise, the replication of the depression-specific finding (RPE hypo-reactivity) in a clinical sample was nice to see.

Weaknesses:

While the anxiety- and depression-specific factors had differential effects on mood variability (Fig 2A-D) and RPE reactivity (Fig 2E-G) in all samples, such that the correlations between the two factors and these mood parameters were significantly different, the anxiety factor was not consistently (significantly) associated with either mood-related parameter across samples. However, the authors resolve anxiety-specific predictive effects when they collapse across datasets. While it is intuitive that achieving a larger effective sample size would afford the power necessary to detect such individual differences, this struck me as a major caveat for this set of results.

The associations the authors observe between the 'common factor' of depression and anxiety and risk-aptitudes tendencies-presumably the alpha (exponent) parameter in a prospect theory-type subjective value model. But where is this analysis explained? (i.e. how was this model formulated and how were risk attitude parameters estimated?) And what is the interpretation of this finding-is there precedent for looking at risk attitudes in this task? And why would these predictive effects only be observed in relation to the common, but not unique factors of anxiety and depression?

Comments on revised version.

I believe the weaknesses identified in the previous round of review have been adequately addressed by the authors, and my suggestions concerning clarity of presentation have by and large been implemented by the authors.

# Author comment: Author response

**DOI:** 10.7554/eLife.110631.3.sa4

**Authors:** Zhihao Wang, Ting Wang, Tian Nan, Jiahua Xu, André Aleman, Yuejia Luo, Bastien Blain, Yunzhe Liu, Pengfei Xu

**Affiliations:** Shenzhen University of Advanced Technology Shenzhen China; South China Normal University Guangzhou China; Beijing Normal University Beijing China; Chinese Institute for Brain Research Beijing China; Maastricht University Maastricht Netherlands; Beijing Normal University Beijing China; University College London London United Kingdom; Beijing Normal University Beijing China; Beijing Normal University Beijing China

The following is the authors’ response to the original reviews.

**eLife Assessment**

This important study uses a tripartite transdiagnostic computational framework to distinguish depression-specific, anxiety-specific, and shared psychopathology dimensions, in their relationships to mood variability and mood reactivity to reward prediction errors across multiple large non-clinical cohorts and a clinical sample. The evidence is convincing overall because the study combines large samples, a well-characterized gambling task and in-depth computational and psychometric analyses, and it replicates the depression-specific association with blunted reward prediction error-sensitivity in a clinical sample. However, the anxiety-specific effects are less consistently supported across individual datasets, may be underpowered in the clinical cohort because of comorbidity, and some aspects of the factor-analytic, risk-attitude, and mediation analyses would benefit from clearer explanation. These findings advance a mechanistic account of how distinct symptom dimensions differentially shape reward-based mood updating and variability, providing a principled framework for future transdiagnostic modeling.

We thank the editors and reviewers for this important assessment.

Regarding inconsistent results for anxiety-related effects in healthy datasets. Although the anxiety-specific factor showed associations in the expected direction across healthy datasets, these associations were not significant in several individual datasets. Specifically, anxiety-specific scores were positively correlated with mood variation (laboratory dataset: r = 0.10, p = 0.531; online dataset 1: r = 0.08, p = 0.026; online dataset 2: r = 0.19, p = 0.004) and with RPE-related mood sensitivity (laboratory dataset: r = 0.04, p = 0.820; online dataset 1: r = 0.05, p = 0.216; online dataset 2: r = 0.19, p = 0.004; Figures 2A–C and 2E–G). This pattern may partly reflect limited statistical power at the single-dataset level. Because these datasets used comparable task and questionnaire procedures and showed positive effect directions, we conducted pooled analyses to obtain a more stable estimate. Importantly, these analyses included dataset as a random intercept in mixed-effects models to account for between-dataset differences. Thus, the pooled analysis provides an integrated estimate across samples, conceptually similar to an individual-participant-data meta-analytic approach. The pooled results provided evidence for the expected anxiety-specific associations with greater mood variability and heightened RPE-related mood sensitivity in non-clinical participants (mood variation: *t* = 3.46, *p* < 0.001; RPE-related mood sensitivity: *t* = 2.60, *p* = 0.009). In addition, we conducted a mini meta-analysis, and results support that anxiety is associated with intensified mood fluctuations and increased mood sensitivity to RPE in non-clinical participants. We have clarified this point below:

Pages 10-11:

“Correlations between the anxiety-specific factor and mood variation were positive in direction across datasets, although they were not statistically significant in several datasets (the laboratory dataset: *r* = 0.10, *p* = 0.531; the online dataset 1: *r* = 0.08, *p* = 0.026; the online dataset 2: *r* = 0.19, *p* = 0.004). Similarly, correlations between the anxiety-specific factor and *β_RPE_* were positive in direction but statistically inconsistent across datasets (the laboratory dataset: *r* = 0.04, *p* = 0.820; the online dataset 1: *r* = 0.05, *p* = 0.216; the online dataset 2: *r* = 0.19, *p* = 0.004; Figure 2A-C & 2E-G). Because these datasets used comparable task and questionnaire procedures and showed positive effect directions, and because reliable individual differences often require large samples to detect[48], we combined the laboratory dataset, online dataset 1, and online dataset 2 (total N = 1,026). This approach is analogous to an individual-participant-data meta-analytic analysis. We fitted linear mixed-effects models predicting mood variation and *β_RPE_* from the three bifactor scores, with dataset included as a random intercept to account for dataset-level variability. For mood variation, the anxiety-specific factor was positively associated with mood variation (*t* = 3.46, *p* < 0.001), whereas the depression-specific factor was negatively associated with mood variation (*t* = -6.13, *p* < 0.001). For RPE-related mood sensitivity, the anxiety-specific factor was positively associated with *β_RPE_* (*t* = 2.60, *p* = 0.009), whereas the depression-specific factor was negatively associated with *β_RPE_* (*t* = -5.30, *p* < 0.001). These associations remained significant after controlling for gender, age, task earnings, and mood drift. In addition, we performed a mini meta-analysis on these correlation coefficients[49]. Results showed significant positive correlation for both mood variation and RPE-related mood sensitivity (mood variation: Z = 3.399, 95 % CI for correlation coefficient r [0.045, 0.166]; RPE-related mood sensitivity: Z = 2.618, 95 % CI for correlation coefficient r [0.021, 0.143]), supporting that anxiety is associated with intensified mood fluctuations and increased mood sensitivity to RPE in non-clinical participants.”

We also admit that the anxiety-related effects were less robust than the depression-related effects and were detectable only in the pooled dataset (n = 1,026); therefore, they require further replication in larger samples.

Page 17:

“Notably, the anxiety-related effects were less robust than the depression-related effects and were detectable only in the pooled dataset (n = 1,026); therefore, they require further replication in larger samples.”

Regarding be underpowered sample size in the clinical cohort. We agree that the clinical sample may have been underpowered to detect anxiety-specific effects, especially given the high comorbidity between anxiety and depression in affective disorders (Table S8). Based on the effect size observed in the non-clinical datasets (*r* = 0.079), we estimated that a sample size of 1,226 would be required to detect this effect with 80% statistical power using a two-tailed test with α = .05. This estimate is substantially larger than the current clinical sample size (*n* = 116). Although these covariate analyses support the robustness of the depression-related effect, they do not resolve whether the absence of the anxiety-related effect reflects limited power or true clinical discontinuity. We also revised the Discussion to explicitly acknowledge that the anxiety-related effect observed in the pooled non-clinical dataset was not replicated in the clinical sample. We now note two possible interpretations. First, this discontinuity may reflect limited statistical power in the clinical sample. Second, and more speculatively, it may reflect a disruption of mood homeostasis in affective disorders (Paulus, 2007). In non-clinical individuals, the counterbalancing associations of depression- and anxiety-related traits with mood variation may contribute to emotional equilibrium. In contrast, affective disorders may involve a loss of this regulatory balance, reducing the ability to stabilize mood in the face of competing depression- and anxiety-related affective signals. We have revised the manuscript as follows:

Pages 12-13:

“To test whether abnormalities in RPE-driven mood fluctuations can serve as clinically relevant computational markers of depression- and anxiety-related symptom dimensions, we recruited patients with affective disorders (n = 116) to complete the same questionnaire battery and gambling task with momentary mood ratings (Figure 1). Demographic, psychological, and clinical characteristics are summarized in Table 1 and Table S8. We observed significant negative correlations between depression-specific scores and both mood variation (*r* = -0.239, *p* = 0.009) and RPE-related mood sensitivity (β_RPE; *r* = -0.216, *p* = 0.020). These associations remained significant after controlling for demographic and clinical covariates, task earnings, and mood drift (*ps* < 0.05). Bootstrap validation yielded consistent results. Mediation analyses further showed that reduced mood sensitivity to RPEs statistically mediated the association between depression-specific scores and lower mood fluctuations (a × b = -0.141, 95% CI = [-0.261, -0.038], *p* = 0.021; Figure 3). However, we did not observe significant correlation with anxiety (mood variation: *r* = -0.092, *p* = 0.327; β_RPE: *r* = -0.095, *p* = 0.311).”

Pages 17-18:

“Notably, the pattern of heightened RPE sensitivity observed in the pooled non-clinical dataset was not observed in the clinical sample. On the one hand, this discontinuity may reflect that the clinical sample was underpowered to detect anxiety-specific effects, especially given the high comorbidity between anxiety and depression in affective disorders (Table S8). Based on the effect size observed in the non-clinical datasets (*r* = 0.079), we estimated that a sample size of 1,226 would be required to detect this effect with 80% statistical power using a two-tailed test with α = .05. This estimate is substantially larger than the current clinical sample size (*n* = 116). On the other hand, it may reflect a disruption of mood homeostasis in clinical populations[41,58]. In non-clinical individuals, counterbalancing associations of depression- and anxiety-related traits with mood variation may help maintain emotional equilibrium. In contrast, affective disorders may involve a loss of such regulatory balance, reducing the ability to stabilize mood in the face of competing depression- and anxiety-related affective signals.”

Abstract:

“Results showed that depression was associated with dampened mood fluctuations due to mood hyposensitivity to RPE. Importantly, this pattern was also found in patients with affective disorders. In contrast, anxiety correlated with heightened mood fluctuations stemming from mood hypersensitivity to RPE in non-clinical participants.”

We have also revised the manuscript accordingly to make the factor-analytic, risk-attitude, and mediation analyses clear.

**Reviewer #1 (Public review):**

Summary:

This is a very interesting paper. The research question is intriguing, allowing the authors to address commonly observed comorbidities between depression and anxiety and their dissociable and opposite relationship to mood fluctuations and sensitivity to reward prediction errors. The computational analyses are very in-depth, including many state-of-the-art checks and validations. Another strength is the inclusion of several large or very large samples, including a patient sample in addition to the general population sample.

I have the following questions:

(1) Factor analysis

I found the hierarchical organization of the factors interesting. While this is a very common procedure in, for example, the field of intelligence (producing sub-scores and a general g factor), it is not yet very commonly used in the field of computational psychiatry (though it has been validated before for anxiety/depression, so it is used here with good reason). I was also impressed by the methodological depth. In particular, it was of note how thoroughly done it was (for example, repeating the EFA on the second half of the data set). I have one question though: is the sample size too small for the exploratory analyses, given the number of items? Given the stability across the half-split, I imagine it is not. Perhaps the authors could spell out how many items, what would be the recommended standard for a subject-to-item ratio, and comment on this. A very technical point, the authors should specify how they extracted the factor scores from the other data sets (is it using the Thurstone or Bartlett method)? From experience (though not doing a hierarchical factor analysis), Bartlett can be somewhat better compared to the default (Thurstone) - better as in the resulting factors more closely recapitulating the factor correlations in the original sample (and independence of responses of other participants in a sample for computing a person's factor score). Could you also comment on similarities or divergences in this hierarchical factor analysis approach from another one recently used transdiagnostically in Wise et al. (2026, Translational Psychiatry)?

We thank the Reviewer for the positive evaluation of our hierarchical factor-analytic approach and for recognizing the methodological depth of our analyses. We are particularly grateful for the Reviewer’s constructive suggestions regarding the participant-to-item ratio, factor score extraction, and the relation between our approach and recent transdiagnostic hierarchical factor-analytic work.

First, hierarchical organization. As the Reviewer noted, hierarchical and bifactor representations have a well-established tradition in intelligence research, where they are used to model the g factor alongside domain-specific abilities (e.g., Reise, 2012; Rodriguez et al., 2016). Crucially, the use of such hierarchical structures in the present study was motivated primarily by theory and evidence from anxiety and depression research, rather than by analogy to intelligence research alone. This tradition can be traced back to the tripartite model of anxiety and depression (Clark & Watson, 1991), which distinguished a broad shared component of general distress or negative affect from more specific anxiety- and depression-related components. Subsequent psychometric work has further supported bifactor and hierarchical representations of anxiety and depression symptoms, including models that separate a general internalizing/distress factor from symptom-specific dimensions (e.g., Simms et al., 2008). More recently, similar hierarchical symptom structures have also been adopted in computational psychiatry to relate shared and specific affective symptom dimensions to task-derived computational parameters (Gagne et al., 2020, 2022; see Wise et al., 2023 for a review). Thus, the bifactor structure used here provides a theoretically motivated way to capture both the variance shared by anxiety and depression and the symptom-specific variance relevant to our computational analyses. We have clarified this point in the revised manuscript as follows:

Pages 3-4:

“Recent work has used bifactor models of the tripartite model of depression and anxiety to clarify their distinct features and differential influences on decision-making[31,32]. The tripartite model of anxiety and depression proposes that these two symptom dimensions share a broad general distress or negative affect component while also including symptom-specific components: low positive affect/anhedonia is more specific to depression, whereas physiological hyperarousal is more specific to anxiety[30,33,34]. Bifactor analysis offers a way to model this structure statistically. In a bifactor model, symptoms load on a general factor reflecting their shared variance and on specific factors capturing residual variance in narrower symptom dimensions after accounting for the general factor. Although bifactor and hierarchical models have long been used in psychometrics, e.g., intelligence research[35,36], their application to anxiety and depression is grounded in the tripartite model and subsequent psychometric work distinguishing general internalizing/distress from symptom-specific dimensions. This framework has recently been extended to computational psychiatry, where shared and specific affective symptom dimensions have been linked to task-derived computational parameters. For example, Gagne et al. (2022) used bifactor analysis to show that depression was associated with weaker prior beliefs, whereas anxiety was associated with a stronger negative bias in belief updatin31.”

Second, the participant-to-item ratio. We agree that the ratio of 450 participants to 128 items in the EFA split-half sample, approximately 3.5:1, is below some conventional sample-size recommendations for exploratory factor analysis, including the often-cited recommendation of five participants per item (Costello & Osborne, 2005). However, as the Reviewer noted, the split-half analysis showed a stable factor structure, and the independent CFA in the other split-half sample further supported the robustness of the solution. Importantly, participant-to-item ratios are only one criterion for evaluating factor recovery. De Winter, Dodou, and Wieringa (2009) demonstrated that reliable EFA solutions may be obtained even with relatively small samples when the data are well-conditioned, such as when factor loadings are high, the number of factors is small, and each factor is defined by multiple items. These conditions were largely met in our data. We have clarified this point in the revised manuscript as follows:

Supplementary Page 3:

“In addition, the ratio of 450 participants to 128 items in the EFA split-half sample, approximately 3.5:1, is below some conventional sample-size recommendations for EFA, including the often-cited recommendation of five participants per item[8]. However, the split-half EFA yielded a stable factor structure, and the independent CFA further supported the robustness of this solution. Moreover, reliable EFA solutions may be obtained even with relatively small samples when the data are well-conditioned, such as when factor loadings are high, the number of factors is small, and each factor is defined by multiple items9. These conditions were largely met in our data.”

Next, factor score extraction. We followed prior work using bifactor modeling in computational psychiatry (Gagne et al., 2020) and extracted factor scores with the Anderson–Rubin method, implemented using psych::factor.scores with method = "Anderson". This approach yields standardized and mutually orthogonal factor scores, which is particularly appropriate for our subsequent correlation analyses because it produces orthogonal scores and therefore avoids multicollinearity among the general, depression-specific, and anxiety-specific factors. In addition, as suggested by the Reviewer, we extracted factor scores using the Bartlett method from an oblique bifactor model, which allowed the depression- and anxiety-specific factors to correlate. In the combined dataset (n = 1,026), anxiety- and depression-specific scores were significantly correlated when extracted using the Bartlett method (*r* = 0.638, *p* < 0.001), whereas, as expected, they were effectively uncorrelated when extracted using the Anderson–Rubin method (*r* < 0.001, *p* = 1.000). This comparison suggests that the Anderson–Rubin method is more appropriate for our analytic aim of estimating the unique associations of shared and symptom-specific components with task-derived parameters, because it separates the general distress/internalizing factor from the statistically separable residual anxiety- and depression-specific components. For this reason, we retained the Anderson–Rubin factor scores in the main analyses. We have clarified this point in the revised manuscript as follows:

Supplementary Page 3:

“For factor score extraction, we followed prior work using bifactor modeling in computational psychiatry[10] and extracted factor scores with the Anderson–Rubin method, implemented using psych::factor.scores with method = "Anderson". This approach yields standardized and mutually orthogonal factor scores, which is particularly appropriate for our subsequent correlation analyses because it avoids multicollinearity among the general, depression-specific, and anxiety-specific factors. As a robustness check, we also extracted factor scores using the Bartlett method from an oblique bifactor model, which allowed the depression- and anxiety-specific factors to correlate. In the combined dataset (n = 1,026), anxiety- and depression-specific scores were significantly correlated when extracted using the Bartlett method (r = 0.638, p < 0.001), whereas, as expected, they were effectively uncorrelated when extracted using the Anderson–Rubin method (r < 0.001, p = 1.000). This comparison suggests that the Anderson–Rubin method is more appropriate for our analytic aim of isolating the unique contributions of shared and symptom-specific variance, because it separates the general distress/internalizing factor from residual anxiety- and depression-specific components. Thus, we retained the Anderson–Rubin factor scores in the main analyses.”

Finally, the similarities and differences between our hierarchical factor-analytic approach and the recent transdiagnostic hierarchical factor-analytic approach of Wise et al. (2026). Both approaches fit EFA models with different numbers of factors and use cross-level correlations to characterize hierarchical symptom structure. However, Wise et al. (2026) applied this framework to a broader symptom battery covering transdiagnostic and neurodevelopmental dimensions, identifying a hierarchy that included a general psychopathology factor and more specific dimensions such as internalizing, externalizing, inattentive/neurodevelopmental, mood/anxiety, and withdrawal. In contrast, our study focused more narrowly on anxiety and depression dimensions, with the goal of deriving symptom factors that could be linked to task-derived computational parameters. Accordingly, whether the current findings are specific to anxiety- and depression-related symptom dimensions or instead reflect broader transdiagnostic psychopathology or nonspecific response-related variance remains unknown. Future studies should include measures covering a wider range of psychiatric dimensions, such as internalizing, externalizing, inattentive/neurodevelopmental, mood/anxiety, and withdrawal dimensions identified by Wise et al. (2026), to better determine whether the links among symptom dimensions, RPE-related mood sensitivity, and mood variability are disorder-specific or transdiagnostic. We have discussed this point in the revised manuscript as follows:Page 19:

“Future studies should include measures covering a wider range of psychiatric dimensions, such as internalizing, externalizing, inattentive/neurodevelopmental, mood/anxiety, and withdrawal dimensions identified by Wise et al. (2026)[59], to better characterize whether links among symptom dimensions, RPE sensitivity, and mood variability are disorder-specific or transdiagnostic.”

(2) Linking factors to task parameters

As I understand it, the authors relate the orthogonalized depression/anxiety to task parameters (sensitivity to RPEs on mood and mood variations) using correlations. In order to have a better understanding of how this relates to other commonly used approaches, I would pose two questions:

(i) What are the correlations when the full (non-orthogonalized) factor scores for depression and anxiety are used? Are the signs the same?

(ii) What are the results when, instead of the independent correlations, the authors perform b_RPE ~ anxiety + depression (again using the non-orthogonalized factors)?I'm assuming all of these analyses should give the same results if the authors' hypothesis of opposing effects of anxiety and depression holds true.

We thank the Reviewer for these helpful comments. Our original analyses used orthogonalized depression- and anxiety-specific factor scores because this approach is aligned with our analytic aim of separating shared and symptom-specific variance within the tripartite/bifactor framework of anxiety and depression (Clark & Watson, 1991), and has been used in prior work (Gagne et al., 2020, 2022; see Wise et al., 2023 for a review). Orthogonalization allows us to statistically separate the shared distress component from the symptom-specific components of anxiety and depression, which was central to our hypothesis regarding their opposing associations with task-derived parameters. As expected, the orthogonalized anxiety- and depression-specific factor scores were uncorrelated in the combined dataset (*r* < 0.001, *p* = 1.000; n = 1,026). By contrast, the full non-orthogonalized depression and anxiety scores retained substantial shared variance and were highly correlated (*r* = 0.638, *p* < 0.001; n = 1,026), making their separate associations less straightforward to interpret.

Nevertheless, we agree that analyses using the full non-orthogonalized depression and anxiety scores provide an important comparison with more commonly used non-orthogonal symptom-score approaches. We therefore conducted the analyses suggested by the Reviewer. When the full depression and anxiety scores were entered separately into linear mixed-effects models predicting RPE-related mood sensitivity, with dataset included as a random intercept, the anxiety association was not significant (anxiety: b = 0.002, *t* = 1.130, *p* = 0.259; depression: b = -0.008, *t* = -3.732, *p* < 0.001). By contrast, when the full non-orthogonalized anxiety and depression scores were entered simultaneously in the same linear mixed-effects model, the original pattern was replicated: anxiety and depression showed opposing associations with RPE-related mood sensitivity (anxiety: b = 0.012, *t* = 4.619, *p* < 0.001; depression: b = -0.017, *t* = -5.851, *p* < 0.001). This pattern is consistent with a mutual suppression effect: shared variance between anxiety and depression may obscure their unique associations when examined separately, whereas the simultaneous regression model reveals their opposing symptom-specific associations.

Together, these supplementary analyses support our original interpretation that RPE-related mood sensitivity is associated with the separable anxiety- and depression-specific components in opposite directions. We have revised the manuscript as follows:

Supplementary Pages 3-4:

“We further analyzed non-orthogonalized full depression and anxiety scores to assess the robustness of our results. When full depression and anxiety scores were entered in separate linear mixed-effects models predicting RPE-related mood sensitivity, with dataset included as a random intercept, the anxiety association was not significant (anxiety: b = 0.002, *t* = 1.130, *p* = 0.259; depression: b = -0.008, *t* = -3.732, *p* < 0.001). By contrast, when the full non-orthogonalized anxiety and depression scores were entered simultaneously in the same linear mixed-effects model, the original pattern was replicated: anxiety and depression showed opposing associations with RPE-related mood sensitivity (anxiety: b = 0.012, *t* = 4.619, *p* < 0.001; depression: b = -0.017, *t* = -5.851, *p* < 0.001). This pattern is consistent with a mutual suppression effect: shared variance between anxiety and depression may obscure their unique associations when examined separately, whereas simultaneous regression reveals their opposing symptom-specific associations. These results support our interpretation that RPE-related mood sensitivity is linked to the separable anxiety- and depression-specific components.”

Minor comments:

(1) The authors should write down when the data were collected for each study. This is because AI capabilities have massively increased since ~2020 in quite specific steps (with the public release of new AI models), meaning that AI is likely to have been able to do tasks and questionnaires without detection if data were collected recently.

We thank the Reviewer for this important comment. We have now added the data collection periods for each dataset in Table 1. The laboratory and clinical dataset were collected in a controlled laboratory setting rather than through online testing. As shown in Table 1, all online experiments were conducted before November 2022, prior to the public release of ChatGPT and its broad entry into public awareness. Therefore, our data were unlikely to have been substantially affected by AI-assisted responding. We have clarified this point in the revised manuscript as follows:

Page 20:

“See Table 1 for demographic information and data collection periods. Because online data collection may raise concerns about AI-generated responses, we note that artificial intelligence tools, such as ChatGPT, became widely known to the public in November 2022, whereas all online experiments in the present study were conducted before November 2022 (see Table 1). Therefore, these data were unlikely to have been substantially affected by participants’ use of AI tools.”

(2) The authors should include a statement in the methods section that checks for AI were done. If none yet, could you do any? Recent papers (Westwood, PNAS 2025; van der Stigchel PNAS, 2026) point to the risk since at least the release of o4-mini (used in the cited paper to create very human-like behaviour).

We thank the Reviewer for this helpful comment. We have clarified this point in the revised manuscript as follows:

Page 20:

“See Table 1 for demographic information and data collection periods. Because online data collection may raise concerns about AI-generated responses, we note that artificial intelligence tools, such as ChatGPT, became widely known to the public in November 2022, whereas all online experiments in the present study were conducted before November 2022 (see Table 1). Therefore, these data were unlikely to have been substantially affected by participants’ use of AI tools.”

(3) It would have been good to collect questionnaires of other, thought to be unrelated psychiatric traits, like compulsivity or schizophrenia symptoms, to check the specificity of the results, also under the assumption that higher scores on either of these skewed questionnaires can pick up individual differences in 'bad questionnaire completion'. The authors should comment on the absence of other questionnaires in the discussion in the limitations section.

We thank the Reviewer for this helpful comment. We agree that the absence of broader psychiatric trait measures limits our ability to evaluate the specificity of the observed associations. Our symptom assessment focused specifically on anxiety and depression because the study was motivated by hypotheses about their potentially opposing links with RPE sensitivity and mood variability. Although previous research has shown intact mood sensitivity to RPEs in individuals with suicidal thoughts and behaviors (Wang et al., 2026), we cannot determine whether the current findings are specific to anxiety- and depression-related symptom dimensions or instead reflect broader transdiagnostic psychopathology or nonspecific response-style variance.

Regarding the concern that higher scores on symptom questionnaires with skewed score distributions may partly capture individual differences in poor-quality questionnaire responding, we note that we implemented strict data-quality procedures for both questionnaire and task data. Four attention-check items were embedded throughout the questionnaire battery, requiring participants to select a prespecified response, for example, “Please select the second option for this item.” Similarly, four attention-check trials were embedded throughout the gambling task. For example, participants were asked to choose between a certain gain of 20 points and a gamble with possible outcomes of 35 and 55 points, for which the dominant response was to choose the gamble option. Participants who failed any of these attention checks were excluded. In addition, our behavioral and mood data reproduced key patterns reported in previous studies (Rutledge et al., 2014 & 2015) using momentary mood ratings during gambling tasks, including higher mood following gains than following losses and systematic mood drift over time (all ps < 0.001). These procedures and validation checks reduce the likelihood that the present findings were driven by poor questionnaire or task completion. We have clarified this point in the revised manuscript as follows:

Page 19:

“Second, our symptom assessment focused specifically on anxiety and depression. This choice was motivated by our primary hypotheses, but it limits our ability to evaluate the specificity of the observed associations. Recent work has shown that individuals with suicidal thoughts and behaviors exhibit reduced mood sensitivity to certain rewards (CR), but not to RPEs[49], suggesting that the current RPE-related effects are not driven by suicide-related processes. However, because we did not assess other psychiatric dimensions, such as compulsivity or schizophrenia-spectrum symptoms, we cannot determine whether the current findings are specific to anxiety- and depression-related symptom dimensions or instead reflect broader transdiagnostic psychopathology or nonspecific response-related variance. Future studies should include measures covering a wider range of psychiatric dimensions, such as internalizing, externalizing, inattentive/neurodevelopmental, mood/anxiety, and withdrawal dimensions identified by Wise et al. (2026)[59], to better characterize whether links among symptom dimensions, RPE sensitivity, and mood variability are disorder-specific or transdiagnostic.”

Page 20：

“Participants were excluded if (1) they failed any of the attentional checks (4 items); (2) they made the same choices for all items; (3) they responded with extreme inconsistency in two similar questionnaires (difference in z-scores out of ±2).”

Page 21:

“There were four items for attentional checks, which required the participants to make a specific choice and were embedded in the entire measurements, e.g., ‘please select the second option for this item’.”

Page 22:

“We also set 4 trials embedded in the entire task for attentional checks. For example, participants were asked to make a choice between a certain gain 20 and a gamble 35/55, where the correct response for this trial was the gamble choice.”

Page 5:

“Choice data (e.g., gambling rates) and mood data (e.g., initial mood, mean mood, and mood variation) showed patterns similar to those reported in previous studies measuring momentary mood during gambling tasks (Figure S2 & S3)[10,45]. We also replicated established effects on momentary mood: mood was higher following gains than following losses, and mood drifted over time (all *ps* < 0.001; Figure S4).”

(4) The authors could include a more explicit sentence in the abstract stating that the anxiety result did not hold up in the clinical population.

We thank the Reviewer for this helpful comment. We have clarified this point in the revised manuscript as follows:

Abstract:

“Results showed that depression was associated with dampened mood fluctuations due to mood hyposensitivity to RPE. Importantly, this pattern was also found in patients with affective disorders. In contrast, anxiety correlated with heightened mood fluctuations stemming from mood hypersensitivity to RPE in non-clinical participants.”

**Reviewer #2 (Public review):**

Summary:

Despite their common co-occurrence, depression and anxiety are known to alter mood fluctuations in opposite ways. Here, the authors aimed at distinguishing depression-specific from anxiety-specific from psychopathology-general effects of reward processing on mood fluctuations, focusing on reward prediction errors (RPEs), which are known to be linked to mood fluctuations. This mechanistic study aims at uncovering the process through which these psychopathologies are associated with mood modulations. The authors were able to appropriately test their hypothesis and obtained results corroborating their conclusions.

This work provides a convincing demonstration of the relevance of computational psychiatry (Huys et al, 2016) and the use of decision neuroscience to shed light on the interplay of anxiety, depression, and mood.

Strengths:

The authors used a tripartite model to distinguish depression vs anxiety, as well as a computational model distinguishing reward expectation (EV in the model) from outcome processing through RPE, which are two sequential cognitive processes.

The manuscript adequately addresses the concerns one would have regarding risk-attitudes and regarding referring to trending statistical results.

Weaknesses:

The sample size of the clinical sample (N=116) may not be sufficient to detect anxiety-specific effects due to the high rate of comorbid anxious depression. It would be beneficial to include the number of MDD vs GAD vs anxious depression diagnoses in the clinical population, as this would likely shine light on the power limitations.

We thank the Reviewer for this helpful comment. We agree that the clinical sample may have been underpowered to detect anxiety-specific effects, especially given the high comorbidity between anxiety and depression in affective disorders (see Table S8 for diagnosis, illness duration, and medication status). Based on the effect size observed in the non-clinical datasets (*r* = 0.079), we estimated that a sample size of 1,226 would be required to detect this effect with 80% statistical power using a two-tailed test with α = .05. This estimate is substantially larger than the current clinical sample size (*n* = 116). Although these covariate analyses support the robustness of the depression-related effect, they do not resolve whether the absence of the anxiety-related effect reflects limited power or true clinical discontinuity.

We also revised the Discussion to explicitly acknowledge that the anxiety-related effect observed in the pooled non-clinical dataset was not replicated in the clinical sample. We now note two possible interpretations. First, this discontinuity may reflect limited statistical power in the clinical sample. Second, and more speculatively, it may reflect a disruption of mood homeostasis in affective disorders (Paulus, 2007). In non-clinical individuals, the counterbalancing associations of depression- and anxiety-related traits with mood variation may contribute to emotional equilibrium. In contrast, affective disorders may involve a loss of this regulatory balance, reducing the ability to stabilize mood in the face of competing depression- and anxiety-related affective signals. We have revised the manuscript as follows:

Pages 12-13:

“To test whether abnormalities in RPE-driven mood fluctuations can serve as clinically relevant computational markers of depression- and anxiety-related symptom dimensions, we recruited patients with affective disorders (n = 116) to complete the same questionnaire battery and gambling task with momentary mood ratings (Figure 1). Demographic, psychological, and clinical characteristics are summarized in Table 1 and Table S8. We observed significant negative correlations between depression-specific scores and both mood variation (*r* = -0.239, *p* = 0.009) and RPE-related mood sensitivity (*β_RPE_*; *r* = -0.216, *p* = 0.020). These associations remained significant after controlling for demographic and clinical covariates, task earnings, and mood drift (*ps* < 0.05). Bootstrap validation yielded consistent results. Mediation analyses further showed that reduced mood sensitivity to RPEs statistically mediated the association between depression-specific scores and lower mood fluctuations (a × b = -0.141, 95% CI = [-0.261, -0.038], *p* = 0.021; Figure 3). However, we did not observe significant correlation with anxiety (mood variation: *r* = -0.092, *p* = 0.327; β_RPE: *r* = -0.095, *p* = 0.311).”

Pages 17-18:

“Notably, the pattern of heightened RPE sensitivity observed in the pooled non-clinical dataset was not observed in the clinical sample. On the one hand, this discontinuity may reflect that the clinical sample was underpowered to detect anxiety-specific effects, especially given the high comorbidity between anxiety and depression in affective disorders (Table S8). Based on the effect size observed in the non-clinical datasets (*r* = 0.079), we estimated that a sample size of 1,226 would be required to detect this effect with 80% statistical power using a two-tailed test with α = .05. This estimate is substantially larger than the current clinical sample size (*n* = 116). On the other hand, it may reflect a disruption of mood homeostasis in clinical populations[41,58]. In non-clinical individuals, counterbalancing associations of depression- and anxiety-related traits with mood variation may help maintain emotional equilibrium. In contrast, affective disorders may involve a loss of such regulatory balance, reducing the ability to stabilize mood in the face of competing depression- and anxiety-related affective signals.”

Abstract:

“Results showed that depression was associated with dampened mood fluctuations due to mood hyposensitivity to RPE. Importantly, this pattern was also found in patients with affective disorders. In contrast, anxiety correlated with heightened mood fluctuations stemming from mood hypersensitivity to RPE in non-clinical participants.”

**Reviewer #3 (Public review):**

Summary:

In this submission, Wang and colleagues jointly examine the association between depression and anxiety symptoms and individuals' affective reactivity to reward prediction errors in Ruttledge et al.'s gambling paradigm. Taking a bifactor approach to anxiety and depression in several non-clinical (and one clinical sample), the authors find that anxiety-specific symptoms relate to over-reactivity of mood to reward prediction errors (RPEs) as well as heightened mood variability, while depression-specific symptoms relate to blunted mood sensitivity to RPEs. These depression- but not anxiety-specific relationships replicated in patient samples.

Strengths:

I was impressed that the data-driven, transdiagnostic approach employed by the authors uncovered specific relationships between anxiety and depression-specific factors and RPE reactivity in a well characterized task and computational model, especially in a non-clinical sample. This sheds new light on how these affective processes may be perturbed-and importantly, in different ways-by anxiety and depression symptoms. Likewise, the replication of the depression-specific finding (RPE hypo-reactivity) in a clinical sample was nice to see.

Weaknesses:

(1) While the anxiety- and depression-specific factors had differential effects on mood variability (Figure 2A-D) and RPE reactivity (Figure 2E-G) in all samples, such that the correlations between the two factors and these mood parameters were significantly different, the anxiety factor was not consistently (significantly) associated with either mood-related parameter across samples. However, the authors resolve anxiety-specific predictive effects when they collapse across datasets. While it is intuitive that achieving a larger effective sample size would afford the power necessary to detect such individual differences, this struck me as a major caveat for this set of results.

We thank the Reviewer for this important comment. Although the anxiety-specific factor showed associations in the expected direction across datasets, these associations were not significant in several individual datasets. Specifically, anxiety-specific scores were positively correlated with mood variation (laboratory dataset: r = 0.10, p = 0.531; online dataset 1: r = 0.08, p = 0.026; online dataset 2: r = 0.19, p = 0.004) and with RPE-related mood sensitivity (laboratory dataset: r = 0.04, p = 0.820; online dataset 1: r = 0.05, p = 0.216; online dataset 2: r = 0.19, p = 0.004; Figures 2A–C and 2E–G). This pattern may partly reflect limited statistical power at the single-dataset level.

Because these datasets used comparable task and questionnaire procedures and showed positive effect directions, we conducted pooled analyses to obtain a more stable estimate. Importantly, these analyses included dataset as a random intercept in mixed-effects models to account for between-dataset differences. Thus, the pooled analysis provides an integrated estimate across samples, conceptually similar to an individual-participant-data meta-analytic approach. The pooled results provided evidence for the expected anxiety-specific associations with greater mood variability and heightened RPE-related mood sensitivity (mood variation: *t* = 3.46, *p* < 0.001; RPE-related mood sensitivity: *t* = 2.60, *p* = 0.009). In addition, we conducted a mini meta-analysis, and results support that anxiety is associated with intensified mood fluctuations and increased mood sensitivity to RPE.

However, we have clarified in the revised manuscript that the anxiety-related effects were less robust than the depression-related effects and require further replication in larger samples.

Pages 10-11:

“Correlations between the anxiety-specific factor and mood variation were positive in direction across datasets, although they were not statistically significant in several datasets (the laboratory dataset: *r* = 0.10, *p* = 0.531; the online dataset 1: *r* = 0.08, *p* = 0.026; the online dataset 2: *r* = 0.19, *p* = 0.004). Similarly, correlations between the anxiety-specific factor and *β_RPE_* were positive in direction but statistically inconsistent across datasets (the laboratory dataset: *r* = 0.04, *p* = 0.820; the online dataset 1: *r* = 0.05, *p* = 0.216; the online dataset 2: *r* = 0.19, *p* = 0.004; Figure 2A-C & 2E-G). Because these datasets used comparable task and questionnaire procedures and showed positive effect directions, and because reliable individual differences often require large samples to detect[48], we combined the laboratory dataset, online dataset 1, and online dataset 2 (total N = 1,026). This approach is analogous to an individual-participant-data meta-analytic analysis. We fitted linear mixed-effects models predicting mood variation and *β_RPE_* from the three bifactor scores, with dataset included as a random intercept to account for dataset-level variability. For mood variation, the anxiety-specific factor was positively associated with mood variation (*t* = 3.46, *p* < 0.001), whereas the depression-specific factor was negatively associated with mood variation (*t* = -6.13, *p* < 0.001). For RPE-related mood sensitivity, the anxiety-specific factor was positively associated with *β_RPE_* (*t* = 2.60, *p* = 0.009), whereas the depression-specific factor was negatively associated with *β_RPE_* (*t* = -5.30, *p* < 0.001). These associations remained significant after controlling for gender, age, task earnings, and mood drift. In addition, we performed a mini meta-analysis on these correlation coefficients[49]. Results showed significant positive correlation for both mood variation and RPE-related mood sensitivity (mood variation: Z = 3.399, 95 % CI for correlation coefficient r [0.045, 0.166]; RPE-related mood sensitivity: Z = 2.618, 95 % CI for correlation coefficient r [0.021, 0.143]), supporting that anxiety is associated with intensified mood fluctuations and increased mood sensitivity to RPE.”

Page 17:

“Notably, the anxiety-related effects were less robust than the depression-related effects and were detectable only in the pooled dataset (n = 1,026); therefore, they require further replication in larger samples.”

(2) The authors observe associations between the 'common factor' of depression and anxiety and risk-attitude tendencies, presumably the alpha (exponent) parameter in a prospect theory-type subjective value model. But where is this analysis explained? (i.e. how was this model formulated and how were risk attitude parameters estimated?) And what is the interpretation of this finding - is there precedent for looking at risk attitudes in this task? And why would these predictive effects only be observed in relation to the common, but not unique, factors of anxiety and depression?

We apologize for the unclear statement. We have added a description of the computational modeling of choice behavior. Please see our revisions below:

Page 13:

“Choice parameters were estimated using an established approach–avoidance prospect theory model[10,45,49], which included loss aversion, domain-specific risk attitude parameters in the gain and loss domains, and value-independent Pavlovian approach and avoidance parameters (see Supplementary Note 7 for details of the computational choice models). In this model, risk attitude was quantified by the exponent parameter α in a prospect-theory-inspired subjective value function. Lower α values reflect greater risk aversion, whereas values closer to or above 1 reflect more linear or risk-seeking valuation.”

Supplementary Pages 8-9:

Note 7: Computational model of gambling choice

To quantify how different events impacted participants’ momentary moods during the gamblingIn line with previous studies[14,15], our choice model space included expected value model (cM1), prospect theory model (cM2)[16], and approach-avoidance prospect theory model (cM3)[14]. For cM2 (Equations 6-9), there were 3 parameters, including risk aversion (α, range: [0.3, 1.3]), loss aversion (λ: [0.5, 5]), and inverse temperature (μ: [0, 10]).

$$\displaystyle U_{\text {gamble}}=0.5\left(V_{\text {gain}}\right)^{\alpha}-0.5 \lambda\left(-V_{\text {loss}}\right)^{\alpha}$$

$$\displaystyle U_{\text {certain}}=\left(V_{\text {certain}}\right)^{a} \text { if } V_{\text {certain}} \geq 0$$

$$\displaystyle U_{\text {certain}}=-\lambda\left(-V_{\text {certain}}\right)^{\alpha} \text {if} V_{\text {certain}}\lt0$$

$$\displaystyle P_{\text {gamble}}=\frac{1}{1+e^{-\mu\left(U_{\text {gamble}}-U_{\text {certain}}\right)}}$$

Where V*_gain_* and V*_loss_* are the objective gain and loss from a gamble, respectively. Please note thatV*_gain_* is 0 in loss trials and V*_loss_* is 0 in gain trials. V*_certain_* is the objective value for the certain option. U*_gamble_* and U*_certain_* denote the subjective utilities of the gamble and the certain option, respectively. Choice probability for gamble (P*_gamble_*) is determined by the softmax rule. Building on cM2, cM3 decomposes the decision process into risk-attitude-driven valuation (e.g., loss and risk aversion) and value-insensitive motivational components (Equations 6-8 & 10-12). That is, choice probability for P*_gamble_* in cM3 is jointly determined by the softmax rule and approach/avoidance parameters (*β_gain_*: [-1, 1], *β_loss_*: [-1, 1]). Approach/avoidance parameters are not applied in mixed trials. Please note that a higher gambling rate does not imply a change in risk attitude per se: it can arise from an increased value-insensitive approach bias even when risk-attitude parameters are comparable between groups. Risk attitude is indeed conceptualized in economics as the curvature of the utility function (i.e., the subjective value) of the objective outcomes, with concave curves associated with risk aversion, and convex curves associated with risk seeking[17,18]. By contrast, the approach or avoidance bias apply to all the value. A possible interpretation of the approach bias is that participant approach the option with the highest possible gain (the lottery) in the gain frame; the avoidance bias would then reflect a tendency to systematically avoid the highest potential losses (the lottery) in the loss frame.

$$\displaystyle P_{\text {gamble}}=\frac{1-\beta_{\text {val}}}{1+e^{-\mu\left(U_{\text {gamble}}-U_{\text {certain}}\right)}}+\beta_{\text {val}} \text {if} \beta_{\text {val}} \geq 0$$

$$\displaystyle P_{\text {gamble}}=\frac{1+\beta_{\text {val}}}{1+e^{-\mu\left(U_{\text {gamble}}-U_{\text {certain}}\right)}} \text {if} \beta_{\text {val}}\lt0$$

$$\displaystyle \beta_{\text {val}}\left\{\begin{array}{cl} \beta_{\text {gain}}, & \text {gain trials}, \\ \beta_{\text {loss}}, & \text {loss trials.} \end{array}\right.$$

Model comparison using BIC revealed that the winning model for each dataset was the approach-avoidance prospect theory model (cM3; mean R^2^ = 0.51 for the laboratory dataset, 0.49 for the online dataset1, 0.54 for the online dataset 2, and 0.40 for the clinical dataset; Table S9).

Please also see our interpretation of this finding below:

Page 18:

“With respect to decision-making, prior literature using risky decision-making tasks without feedback has linked pathological anxiety to greater risk aversion[58]. In line with this, our results from a risky decision-making task with feedback suggest that the common factor, rather than anxiety-specific variance per se, is more consistently associated with risk aversion. This suggests that heightened gain-domain risk aversion may be a transdiagnostic feature of internalizing psychopathology, rather than being uniquely attributable to anxiety.”

**Recommendations for the authors:**

**Reviewer #1 (Recommendations for the authors):**

Thank you very much for giving me the opportunity to review this very interesting paper.

Recommendations:

(1) Add more specific ethics information than "study was approved by ethics committee of Beijing normal university".

We thank the Reviewer for this important comment. We have added approval number. Please see our revision below:

Page 20:

“The study was approved by the Ethics Committee of Beijing Normal University (approve number: ICBIR_A_0016_028). Written or electronic informed consent was obtained from all participants before participation.”

(2) Add information on how participants were recruited. I think the websites listed only hosted the experiment/questionnaires?

We thank the Reviewer for pointing this out. We have revised the relevant text as follows:

Page 20:

“A total of 2634 participants via online platforms (questionnaires from https://www.wjx.cn and tasks from https://www.naodao.com) took part in five experiments, including a psychometric experiment, a laboratory experiment, two online replication experiments. Participants were recruited through participant pools and study advertisement. For online experiments, interested participants accessed the study through an online link and completed the questionnaires and task remotely. For the laboratory experiment, participants completed the study in a controlled laboratory setting.”

(3) Typo in Figure 1A, grey panel - psychometric.

We apologize for the typo. We have corrected typographical errors throughout the manuscript.

(4) In the Discussion, there is a section on r-to-z transformations, and I was not quite sure what in the Results this links to.

We thank the Reviewer for pointing out the unclear statement. Please see our revision below:

Page 18:

“First, although anxiety- and depression-related associations differed consistently, the anxiety-specific associations themselves were less robust across datasets.”

**Reviewer #2 (Recommendations for the authors):&&

The Results sections 2 (depression) and 3 (anxiety) could be improved by reducing the back and forth between factors throughout the results. It may be useful to split them into 3 sections: depression only, anxiety only, and depression vs anxiety.

We thank the Reviewer for this helpful suggestion. As suggested, we have reorganized this part into three sections: depression, anxiety, and depression versus anxiety. Please see our revision below:

Page 12:

“Differential associations of depression and anxiety with mood fluctuations. To directly test whether depression- and anxiety-specific factors differed in their associations with mood dynamics, we compared the corresponding correlations. These comparisons showed that depression-specific associations were significantly more negative than anxiety-specific associations for both mood variation (laboratory dataset: *Z* = -1.84, *p* = 0.033; online dataset 1: *Z* = -5.36, *p* < 0.001; online dataset 2: *Z* = -3.42, *p* < 0.001) and β_RPE (laboratory dataset: *Z* = -1.77, *p* = 0.038; online dataset 1: *Z* = -3.67, *p* < 0.001; online dataset 2: *Z* = -4.00, *p* < 0.001; Figures 2A–C and 2E–G). These results support distinct associations of depression- and anxiety-specific factors with RPE-related mood dynamics.”

In the discussion, the authors could have mentioned the brain areas most likely to be involved in these processes, both cognitive and psychopathological, as previous studies (such as Cecchi et al, 2022) have aimed at identifying regions involved in RPE processing while modulating mood in health. A short section on this would be useful to the neuropsychiatric community.

We thank the Reviewer for this helpful suggestion. We agree that the Discussion would benefit from a more explicit consideration of the neural systems that may support RPE-related mood updating and their relevance to psychopathology. We have revised the Discussion accordingly, as shown below:

Page 16:

“Although the present study did not include neuroimaging, the observed computational dissociation may map onto partially distinct neural systems involved in reward learning, mood updating, and affective psychopathology. RPE processing has been consistently linked to striatal–midbrain dopaminergic reward-learning circuits[8,50]. The integration of these reward-learning signals into subjective mood and value-based decision-making may further involve the ventral medial prefrontal cortex and orbitofrontal cortex[44]. In addition, the anterior insula may be particularly relevant for integrating feedback-related signals with affective and interoceptive states[8,44], potentially linking RPE processing to anxiety- and depression-related mood dynamics. Consistent with this view, Cecchi et al. (2022)[51] used intracranial EEG to show that feedback-related neural activity tracks mood fluctuations and risky choice. Future neuroimaging studies should test whether depression-related reductions and anxiety-related increases in RPE-related mood sensitivity are associated with altered interactions among striatal, prefrontal, and insular circuits.”

**Reviewer #3 (Recommendations for the authors):**

(1) The authors need to present a clearer definition of the terms "bifactor analysis" and "tripartite model" in the Introduction. What does tripartite mean in this context? What are the assumptions of such bifactor analyses (e.g. as used in Gagne et al. and the present work) and how, in broad strokes, are they carried out? These are important constructs to clarify for readers outside the computational psychiatry niche.

We thank the Reviewer for this helpful suggestion. We have revised the Introduction accordingly, as shown below:

Page 3:

“Recent work has used bifactor models of the tripartite model of depression and anxiety to clarify their distinct features and differential influences on decision-making[31,32]. The tripartite model of anxiety and depression proposes that these two symptom dimensions share a broad general distress or negative affect component while also including symptom-specific components: low positive affect/anhedonia is more specific to depression, whereas physiological hyperarousal is more specific to anxiety[30,33,34]. Bifactor analysis offers a way to model this structure statistically. In a bifactor model, symptoms load on a general factor reflecting their shared variance and on specific factors capturing residual variance in narrower symptom dimensions after accounting for the general factor.”

(2) Previous examinations of depression and RPE reactivity in this task paradigm, as the authors note (e.g. Rutledge et al., 2017), observed that individuals diagnosed with depression showed an intact association between RPEs and mood. In other words, there was no previously observed relationship between depression and affective reactivity to RPEs in this task context. Here, the authors find that the "unique" depression factor identified by the authors (in a non-clinical sample) is associated with blunted RPE sensitivity - this is worth commenting on specifically.

We thank the Reviewer for this helpful suggestion. We have discussed this point in the Discussion. Please also see it below:

Pages 15-16:

“Our computational model not only replicates the important role of RPEs in mood dynamics but also highlights the divergent mediating roles of RPE-related mood sensitivity in the associations of depression and anxiety with mood fluctuations. The opposite associations of depression and anxiety with mood sensitivity to RPEs complement previous findings of apparently intact RPE-related mood sensitivity in depression[12,25,37]. These findings further underscore the necessity of decomposing shared and specific components of depression and anxiety in studies of mood dynamics, which can enhance our understanding of their distinct associations with emotion processing and cognitive flexibility. This point is consistent with bifactor-based work showing that shared and specific symptom dimensions can have different computational correlates. For example, Gagne et al. (2020) showed that bifactor-derived symptom dimensions differentially relate to maladaptation to environmental volatility[32], complementing previous findings that trait anxiety is associated with inflexible adjustment to volatility[32].”

(3) There is a note (line 247) about the interpretation of the correlations in Figure 2, which attempts to explain away the inconsistent relationships between the anxiety-specific factor and mood variability as well as RPE reactivity observed in Figure 2. I can't say I understand the authors' point here about "signs of positive correlations", so I would say the authors need to clarify their logic here. More to the point, the authors only resolve anxiety-specific predictive effects when they collapse across these datasets. As discussed above (see 'weaknesses'), this is a serious limitation in my view and needs to be discussed as such in the paper.

We apologize for the unclear statement. Although the anxiety-specific factor showed associations in the expected direction across datasets, these associations were not significant in several individual datasets. Specifically, anxiety-specific scores were positively correlated with mood variation (laboratory dataset: r = 0.10, p = 0.531; online dataset 1: r = 0.08, p = 0.026; online dataset 2: r = 0.19, p = 0.004) and with RPE-related mood sensitivity (laboratory dataset: r = 0.04, p = 0.820; online dataset 1: r = 0.05, p = 0.216; online dataset 2: r = 0.19, p = 0.004; Figures 2A–C and 2E–G). This pattern may partly reflect limited statistical power at the single-dataset level.

Because these datasets used comparable task and questionnaire procedures and showed positive effect directions, we conducted pooled analyses to obtain a more stable estimate. Importantly, these analyses included dataset as a random intercept in mixed-effects models to account for between-dataset differences. Thus, the pooled analysis provides an integrated estimate across samples, conceptually similar to an individual-participant-data meta-analytic approach. The pooled results provided evidence for the expected anxiety-specific associations with greater mood variability and heightened RPE-related mood sensitivity (mood variation: *t* = 3.46, *p* < 0.001; RPE-related mood sensitivity: *t* = 2.60, *p* = 0.009). We have revised it to make it clear. Please see our revisions below:

Pages 10-11:

“Correlations between the anxiety-specific factor and mood variation were positive in direction across datasets, although they were not statistically significant in several datasets (the laboratory dataset: *r* = 0.10, *p* = 0.531; the online dataset 1: *r* = 0.08, *p* = 0.026; the online dataset 2: *r* = 0.19, *p* = 0.004). Similarly, correlations between the anxiety-specific factor and β_RPE were positive in direction but statistically inconsistent across datasets (the laboratory dataset: *r* = 0.04, *p* = 0.820; the online dataset 1: *r* = 0.05, *p* = 0.216; the online dataset 2: *r* = 0.19, *p* = 0.004; Figure 2A-C & 2E-G). Because these datasets used comparable task and questionnaire procedures and showed positive effect directions, and because reliable individual differences often require large samples to detect, we combined the laboratory dataset, online dataset 1, and online dataset 2 (total N = 1,026). This approach is analogous to an individual-participant-data meta-analytic analysis while accounting for dataset-level variability. Because these datasets used comparable task and questionnaire procedures and showed positive effect directions, and because reliable individual differences often require large samples to detect[48], we combined the laboratory dataset, online dataset 1, and online dataset 2 (total N = 1,026). This approach is analogous to an individual-participant-data meta-analytic analysis while accounting for dataset-level variability. We fitted linear mixed-effects models predicting mood variation and *β_RPE_* from the three bifactor scores, with dataset included as a random intercept. For mood variation, the anxiety-specific factor was positively associated with mood variation (*t* = 3.46, *p* < 0.001), whereas the depression-specific factor was negatively associated with mood variation (*t* = -6.13, *p* < 0.001). For RPE-related mood sensitivity, the anxiety-specific factor was positively associated with *β_RPE_* (*t* = 2.60, *p* = 0.009), whereas the depression-specific factor was negatively associated with *β_RPE_* (*t* = -5.30, *p* < 0.001).”

Page 17:

“Notably, the anxiety-related effects were less robust than the depression-related effects and were detectable only in the pooled dataset (n = 1,026); therefore, they require further replication in larger samples.”

(4) I expected to see that the authors would also investigate relationships between anxiety/depression related factors and the decay (gamma) parameter in the 'Happiness equation', which is presumably estimated from the data here. While I don't have a strong intuition about directions of (or presence of) predictive relationships here, doesn't it stand to reason that different aspects of psychopathology examined here might map onto how long- (versus short-) lasting the effects of, say, RPEs are, upon mood?

We thank the Reviewer for this important comment. In the healthy datasets, we fitted a linear mixed-effects model predicting the decay parameter (γ) from the three bifactor scores, with dataset included as a random intercept. None of the factors showed a significant association with γ (common: *t* = 0.708, *p* = 0.479; anxiety: *t* = 0.564, *p* = 0.573; depression: *t* = 1.146, *p* = 0.252). In the clinical dataset, we fitted a linear model predicting γ from the three bifactor scores and again found no significant associations (common: *t* = -0.036, *p* = 0.972; anxiety: *t* = -0.047, *p* = 0.963; depression: *t* = 0.052, *p* = 0.959). We have clarified this point in the revised manuscript as follows:

Supplementary Page 5:

“In the healthy datasets, we conducted a linear mixed-effect model against decay parameter (gamma) with all three factors, with dataset as a random factor. Results did not show significant effect (common: *t* = 0.708, *p* = 0.479; anxiety: *t* = 0.564, *p* = 0.573; depression: *t* = 1.146, *p* = 0.252). In the clinical dataset, we conducted a linear model against decay parameter (gamma) with all three factors and found no significant effect (common: *t* = -0.036, *p* = 0.972; anxiety: *t* = -0.047, *p* = 0.963; depression: *t* = 0.052, *p* = 0.959).”

(5) The rationale for and interpretation of the mediation model, which presumably aims to explain the relationships between anxiety- and depression-specific factors, RPE reactivity, and mood variability was barely explained by the authors. At present, I'm not sure what the added value of this analysis is. The authors should either remove or explain/motivate the mediation more clearly.

We thank the Reviewer for this helpful comment. The rationale for the mediation analysis is that mood variability in the task is not only a descriptive behavioral outcome, but may also arise from the degree to which momentary mood is updated by RPEs. Therefore, if depression is associated with reduced RPE-related mood sensitivity and anxiety with increased RPE-related mood sensitivity, these alterations should statistically account for their opposite associations with mood variability. The mediation model directly tested this possibility by examining whether RPE-related mood sensitivity accounted for the association between symptom-specific factors and mood variability. We have clarified this rationale in the revised manuscript as follows:

Page 10:

“Given the strong correlation between β_RPE_ and mood variation (*r*s > 0.67, *p*s < 0.001), we further conducted a mediation analysis to examine whether individual differences in RPE-related mood sensitivity statistically accounted for the association between depression loading and mood variation. This analysis was motivated by the hypothesis that depression-related dampening of mood variability may arise, at least in part, from reduced mood sensitivity to RPEs.”

(6) This submission would benefit from extensive English language copy editing. There are many passages in the paper (in fact, too many to list here) that suffer from either grammatical errors or clarity issues.

We apologize for these mistakes. We have corrected the typographical errors throughout the manuscript.
